# Culture and group-functional punishment behaviour

**DOI:** 10.1017/ehs.2022.32

**Published:** 2022-08-01

**Authors:** Antonio M. Espín, Pablo Brañas-Garza, Juan F. Gamella, Benedikt Herrmann, Jesús Martín

**Affiliations:** 1 Departamento de Antropología Social, Universidad de Granada, Campus de Cartuja S/N, 18071, Granada, Spain; 2 Loyola Behavioral Lab, Universidad Loyola Andalucía, Escritor Castilla Aguayo, 4, 14004 Córdoba, Spain; 3 Centre for Decision Research and Experimental Economics, University of Nottingham, Sir Clive Granger Building, University Park, Nottingham NG7 2RD, UK; 4 Facultad de CC. Económicas y Empresariales, Universidad de Granada, Campus de Cartuja S/N, 18071, Granada, Spain

**Keywords:** Cooperation, punishment, Gypsy/Roma, culture, evolution

## Abstract

Humans often ‘altruistically’ punish non-cooperators in one-shot interactions among genetically unrelated individuals. This poses an evolutionary puzzle because altruistic punishment enforces cooperation norms that benefit the whole group but is costly for the punisher. One key explanation is that punishment follows a social-benefits logic: it is eminently normative and group-functional (drawing on cultural group selection theories). In contrast, mismatch-based deterrence theory argues that punishment serves the individual-level function of deterring mistreatment of oneself and one's allies, hinging upon the evolved human coalitional psychology. We conducted multilateral-cooperation experiments with a sample of Spanish Romani people (*Gitanos* or *Calé*) and the non-*Gitano* majority. The *Gitanos* represent a unique case study because they rely heavily on close kin-based networks and display a strong ethnic identity. We find that *Gitano* non-cooperators were not punished by co-ethnics in only-*Gitano* (ethnically) homogeneous groups but were harshly punished by other *Gitanos* and by non-*Gitanos* in ethnically mixed groups. Our findings suggest the existence of culture-specific motives for punishment: *Gitanos*, especially males, appear to use punishment to protect their ethnic identity, whereas non-*Gitanos* use punishment to protect a norm of universal cooperation. Only theories that consider normative, group-functional forces underlying punishment behaviour can explain our data.

## Graphical abstract

**Figure:**
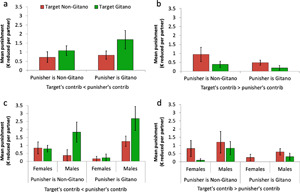

**Social media summary:** An economic experiment with Spanish Roma (*Gitanos*) reveals the existence of culture-specific motives for punishment.

## Introduction

Humans possess an extraordinary capacity for large-scale cooperation and this stands as a theoretical puzzle across the biological and behavioural sciences. Kin selection and direct and indirect reciprocity have been proposed as explanations for the evolution of cooperation in relatively small populations (Hamilton, 1964; Nowak, 2006). To explain prosocial behaviour in large modern societies, however, kinship or reciprocity mechanisms seem to be insufficient because cooperation is observed in ephemeral encounters among unrelated individuals, e.g. in voting, driving, paying taxes, recycling, market interactions and warfare (Boyd & Richerson, 1988; Gintis et al., 2003). For example, maintaining a system in which individual reputation is known by every other individual entails cognitive and monitoring costs which greatly increase with group size, thus limiting the effectiveness of indirect reciprocity to explain large-scale cooperation (Panchanathan & Boyd, 2003; Henrich & Muthukrishna, 2021).

Decentralised (peer) punishment of free-riders has been shown to be a crucial element for understanding cooperation beyond kinship and small-scale groups (Gintis et al., 2003; Boyd et al., 2003; Henrich, 2004; Sigmund, 2007). Punishment is considered altruistic (in the biological sense) when the absolute benefits triggered by the enforcement of the cooperative norm are received by individuals other than the punisher, who pays the cost of punishing (Fehr & Gächter, 2002).

Even if groups in which peer punishment is allowed can outcompete those in which it is not owing to the discouragement of free-riding (Gächter et al., 2008; Sääksvuori et al., 2011; but see Herrmann et al., 2008), altruistic punishers are condemned to a lower evolutionary success within their group (Dreber et al., 2008). It turns out that the provision of a sanctioning system to prevent free-riding can be considered as a second-order social dilemma where individual and collective interests are in conflict (Fehr & Gächter, 2002). Nevertheless, altruistic punishment is frequently observed in controlled experiments with unrelated human subjects, even in one-shot anonymous interactions (Fehr & Gächter, 2002; Henrich et al., 2006; Gächter & Herrmann, 2009; Espín et al., 2012). In fact, the neurobiological evidence suggests that people suffer disutility from observing uncooperative behaviours and derive pleasure from punishing wrongdoers (de Quervain et al., 2004; Crockett et al., 2013), which facilitates punishment decisions, even if they are costly. Yet the evolutionary basis of punishment behaviour and its psychological underpinnings is subject to debate. Why do people pay irrecoverable costs to punish others?

Following cultural group selection (CGS) theories and their associated ‘norm-psychology’ perspective (Soltis et al., 1995; Gintis et al., 2003; Boyd et al., 2003; Henrich, 2004, 2015; Henrich et al., 2010; Chudek & Henrich, 2011; Richerson et al., 2016; Handley & Mathew, 2020; Henrich & Muthukrishna, 2021), those proximate mechanisms are particularly suited for solving the second-order dilemma of decentralised punishment in modern large-scale societies where one-shot interactions with non-relatives are common. Altruistic punishment would have been shaped following a complex process in which genes and culture co-evolve, with cultural adaptation being much more rapid than genetic adaptation.

Under this account, different cultural groups develop the human ‘norm-psychology’ (Chudek & Henrich, 2011) differently in competition with other cultural groups. In particular, specific social behaviours which are advantageous for the group during intergroup competition are transmitted across individuals through social learning mechanisms (i.e. payoff- or frequency-biased imitation) and enforced through sanctions. Cultural groups with more group-beneficial norms, and consequently such norms themselves, are more likely to proliferate. Behavioural variation would not be the result of current ecology alone, as implied by mismatch-related theories (see below), but of its interaction with cultural history as well (thus, not denying evolutionary mismatch). Therefore, it is likely that some cultural groups use decentralised punishment of free-riding extensively, particularly those large groups in which other mechanisms such as kinship, collective/centralised sanctions, ostracism, and reputation are insufficient, while others are more lenient or most probably use it to enforce different norms in a group-functional manner (Henrich et al., 2010; Richerson et al., 2016; Mathew, 2017; Henrich & Muthukrishna, 2021). The collection of ultimate and proximate arguments within this tradition will be referred to as the ‘*social-benefits*’ approach because of its focus on the normativity and group-functional logic of punishment (and because CGS might be a somewhat slippery term; Micheletti, 2020; Smith, 2020). Broadly, this account predicts that punishment will be used to protect key ingroup norms and potentially harm outgroups, as both behaviours can contribute to the success of one's own cultural group in competition with others (Rusch, 2014).

Note that intergroup competition in cultural evolution is a necessary condition for prosocial norms, rather than any other norms or behaviours, to evolve under CGS (Richerson et al., 2016; Henrich & Muthukrishna, 2021). This is due to the existence of multiple evolutionarily stable equilibria, with only some equilibria being ‘prosocial’. Punishment, reputation, and other within-group mechanisms can indeed stabilise any behaviour such as food taboos, clothing customs, and even maladaptive practices like female genital cutting (Boyd & Richerson, 1992; Henrich & Muthukrishna, 2021). It is competition between groups that acts to select those norms which are more beneficial for the cultural group. However, between-group competition does not need to imply extinction, or even intergroup violent conflict (although it is key in human history; Bowles, 2006; Choi & Bowles, 2007); there are other forms of intergroup competition such as prestige-biased group transmission and differential group reproduction/migration without conflict (Richerson et al., 2016; Henrich & Muthukrishna, 2021).

Thus, any valid theory of the emergence of altruistic punishment must explain why sanctions should target those who fail to follow group norms and not others (Henrich, 2015; Henrich & Muthukrishna, 2021). This excludes from the discussion those within-group mechanisms that lead to future benefits for the punisher owing to others’ positive perceptions about punishment behaviour. More specifically, theories based on punisher's (positive) reputation, signaling or partner choice (Raihani & Bshary, 2015, 2019; Jordan et al., 2016; Jordan & Rand, 2020; Batistoni et al., 2022) must explain first why altruistic punishers should have a good reputation or be preferred as partners if it is not because their behaviour benefits the group and its social norms (Henrich & Muthukrishna, 2021). These theories are therefore potential complements rather than substitutes for the social-benefits account of punishment.

However, there are competing accounts that can indeed explain the existence of altruistic punishment without invoking norm-based or equilibrium selection mechanisms. The ‘big mistake’ (or ‘mismatch’) tradition in evolutionary psychology (Cosmides & Tooby, 1992; Lehmann et al., 2007; Krasnow et al., 2012; Delton & Krasnow, 2017) holds that the psychological mechanisms underlying group-beneficial behaviours, such as altruistic punishment, evolved in a period of human history in which nearly all social interactions were repeated and took place among close relatives. Individual selection mechanisms would lie behind the evolution of punishment, which emerged because under those circumstances punishing others benefits the individual's (direct or indirect) inclusive fitness, for instance, by reducing future exploitation by others. Such pan-human social psychology, so the argument goes, misfires in the behaviour of modern humans, who ‘mistakenly’ punish others even in one-shot interactions with unrelated individuals (i.e. where it is no longer adaptive or fitness enhancing). Accordingly, human social psychology is programmed to differentiate between acquaintances and strangers *only* owing to a desire to cultivate and maintain individually profitable, coalitional social-exchange relationships. Thus, the key elements to explain social behaviour according to this line of argument are (coalitional) closeness and genetic relatedness. Different ecologies or environmental cues, however, would lead to different expressions of the common evolved psychology and thus create behavioural variation.

Along this tradition, the most recent and appealing counter-explanation for the emergence of punishment stresses its deterrence logic (e.g. Krasnow et al., 2012, 2016; Delton & Krasnow, 2017). The advocates of *deterrence theory* argue that the main function of punishment is to deter wrongdoers not only from potential future harmful acts against the self but also from harmful acts against valued others – where ‘valued’ means that the punisher's fitness is dependent upon them for genetic or coalitional reasons (Krasnow et al., 2016; Delton & Krasnow, 2017). In intergroup encounters, ingroups should be more valued than outgroups and punishment will tend to be used to defend them from poor treatment (actually, to defend the punisher's interests linked to the ingroups), because harming one's own allies predicts future mistreatment of oneself. Therefore, the presence of ingroups and outgroups is expected to evoke human coalitional psychology and lead to particular patterns of punishment behaviour triggered by cues of potential future damage of the punisher's interests.

This account has been argued to explain results from third-party punishment experiments with group identities in a more parsimonious manner than the social-benefits approach, that is, without any need for norm-based or group-level considerations (Delton & Krasnow, 2017). A typical result from third-party punishment experiments is that an outgroup's wrongdoing towards an ingroup is punished more than an ingroup's wrongdoing (Bernhard et al., 2006; Goette et al., 2006; Jordan et al., 2014; Schiller et al., 2014; Delton & Krasnow, 2017), which challenges a somewhat radical interpretation of the social-benefits perspective. As opposed to other frameworks such as the public goods game, however, in the third-party punishment game it is difficult to disentangle moralistic from competitive/spiteful motives for punishment as the punisher's disposition to cooperate is by design not assessed. A recent study highlights the competitive nature of the third-party punishment of outgroup wrongdoers by showing that it (but not the punishment of ingroup wrongdoers) is harsher when intergroup competition is primed and the cost-to-impact ratio is low so that punishing increases the punisher's relative standing (Guo et al., 2020). The commonly observed ‘intergroup bias’ in third-party punishment can thus be interpreted from a more comprehensive social-benefits lens as combining two elements: (a) the competitive punishment of formidable outgroups may increase the ingroup's relative standing; and (b) an evolved perception that ingroups are morally superior to outgroups (Tajfel, 1974; Brewer, 1999; Rusch, 2014), fitting in perfectly with CGS theories, may lead to punish outgroups’ uncooperativeness relatively more in cultural groups in which universal cooperation is a key norm (for example, ingroups’ free-riding can more likely be perceived as unintentional errors of otherwise cooperative individuals, thus deserving less punishment).

To shed light on the nature of altruistic punishment, we conducted a series of lab-in-the-field public goods experiments with a unique sample of Spanish Romani people (*Gitanos*, also referred to as *Calé*). Romani groups represent the largest ethnocultural minority in Europe. Nonetheless, they have received little attention in experimental research. We are aware of only two studies analysing the behaviour of Romani people: Brañas-Garza et al. (2006) using the ultimatum game and Martín et al. (2019) using time discounting tasks. Behaviour towards Romani people, but not their own behaviour, is studied in Bauer et al. (2018). Our experiments were primarily designed to explore how the ethnic composition of a cooperating group influences peer punishment and to test key elements of the social-benefits account. To do so, we brought together non-*Gitano* and *Gitano* people who, owing to the cultural characteristics outlined below, could help answer important questions about the underpinnings of punishment behaviour.

*Gitanos* constitute a paradigmatic case study for the purposes of this paper because: (a) kinship is at the core of their social life and organisation even if their lifestyle resembles that of their non-*Gitano* neighbors (i.e. the majority Spanish population) in many other aspects. Indeed, consanguinity rates within *Gitano* communities in the geographic area of the study are among the highest ever reported in Europe (Gamella, 2020), at the upper bound of the range observed in traditional small-scale societies of hunter–gatherers and horticulturalists, which are considered to resemble the living conditions of ancestral humans (see Text S1 in the Supplementary Materials). *Gitanos* therefore constitute an exceptionally ‘rare’ case. (b) *Gitanos* display a strong sense of ethnic identity although in many ways they share a bicultural identity (Benet-Martínez et al., 2002). While they mostly speak the majorities’ languages and have adopted the religion and even a number of their neighbors’ mores, they also maintain a strong and vibrant sense of themselves as a separate people. *Gitanos* try to preserve a separate ethnic identity, often reinventing their processes of differentiation, which are mainly based on reproductive strategies where specific factors including marriage, gender and kin systems are crucial (Gay Blasco, 1999; Martín & Gamella, 2005; Gamella & Martín, 2007). As a consequence, for example, even though *Gitanos* and non-*Gitanos* have cohabited the study area for more than 15 generations, mixed marriages have been traditionally rare (less than 5% for over two centuries in the study area and approaching 10% only very recently; Gamella & Álvarez-Roldán, 2021). *Gitanos* and other European Romani groups (but not all) may constitute exceptional examples of ethnic resistance and integration at the same time. Interestingly, recent advances suggest that societies with more intensive kin-based institutions tend to display a stronger ethnocultural identity, ingroup–outgroup differentiation and ingroup loyalty (Schulz et al., 2019; Henrich, 2020).

*Gitanos*, as other Romani groups, have developed a series of autonomous law-making processes that are often encoded in open-ended codes of norms, the *Gitano* Law. Although somewhat less elaborated than in Eastern European Romani groups (Weyrauch, 2001; Marushiakova & Popov, 2007), these processes are important in the effort to limit the escalation of conflicts between families and kin networks, where the duty to defend and support family members is a central concern (San Román, 2010).

There also exist fundamental differences in gender roles between *Gitanos* and non-*Gitanos*. In particular, although this is also changing in recent years, the most relevant difference for the focus of this paper is that *Gitano* norms prescribe women to assume a secondary role in the presence of males in public encounters (Gay Blasco, 1999; Gamella, 2000; Gamella & Martín, 2007; San Román, 2010), whereas normative principles of this type are not observed among non-*Gitanos*. See Text S1 for more ethnographic details.

## Basic design and hypotheses

Before putting forward our research questions and hypotheses, we summarise the basic elements of the experimental design. We conducted our experiments with a total of 320 participants (mean age = 42.80 ± 18.42 SD, 59% females). We recruited *Gitano* and non-*Gitano* people from five small semi-rural towns with a large *Gitano* population in southern Spain. Participants played a one-shot public goods game with peer punishment (PGP) involving real monetary stakes in anonymous four-person groups. Given that participants only played one round of the game and groups were formed anonymously, no strategic concerns (e.g. about potential consequences in future interactions) were present for punishment decisions.

The experimental design comprises two conditions (between-subjects): participants played the PGP in either (a) *homogeneous* groups composed of either only *Gitanos* or only non-*Gitanos* or (b) *mixed* groups with two *Gitano* and two non-*Gitano* members. Importantly, the two conditions were run in different sessions. Thus, ethnic identity was made particularly salient in the mixed sessions because in the homogenous sessions there were only members of one's own cultural group (Tajfel, 1974; Brewer, 1999; Dovidio et al., 2008). While among minority status groups, such as *Gitanos*, group identity is typically carried to every public environment (Pinel, 1999), in the mixed condition the behaviour of the two cultural groups could be directly compared by the participants, which should enhance the salience of intergroup encounter cues and hence of ethnic identity. Still, *Gitanos* and non-*Gitanos*, as minority and majority status groups, experience this ethnocultural difference asymmetrically (with asymmetries of power, position and perspective, as well as more subtle experiential and interactional asymmetries; Brubaker et al., 2018).

Following standard procedures (Gächter & Herrmann, 2009), participants in the PGP first made their cooperation decisions by means of (anonymously) allocating money from their €10 endowment to a group pot. Contributions were doubled and evenly shared among the four group members. Thus, the more one contributes to the group pot (i.e. the public good), the larger the total group benefit, but the lower the decision maker's personal benefit, all else equal. This creates the classical social dilemma between collective and individual interests.

After all the participants had made their decisions, they were shown the contributions of each of the other three group members and allowed to spend part of their earnings in order to reduce others’ earnings (punishment stage): €1 spent on punishment reduced the target individual's earnings by €3. Note that participants contributed knowing beforehand that they could be punished, which introduces strategic incentives to cooperate in order to avoid being punished. The reasons underlying contribution decisions are multiple and, therefore, cooperation does not constitute the main focus of our study. By means of a subtle procedure which preserved participants’ anonymity, we allowed participants in the mixed groups to match the ethnicity and contributions of each of the other three group members. Hence, our procedure let participants condition their punishment decisions on the target's ethnic identity (this was not relevant in the homogenous groups since all four members were of the same cultural group). See the Methods for more details.

### Research questions and hypotheses

Our experiments allow us to test a series of hypotheses about our participants’ punishment behaviour based on past empirical and theoretical evidence and our own ethnographic work (see Text S1). To build these hypotheses, the main variables we consider are ethnicity, treatment condition and gender. Whenever possible, we compare predictions built upon social-benefits arguments with predictions emanating from competing approaches, in particular the mismatch-based deterrence theory. As mentioned, we test the norm-psychology account inherent to the social-benefits approach by highlighting key differential cultural norms of *Gitanos* and non-*Gitanos* observed in our ethnographic work in the study area. This account states that human social psychology is unique in the animal kingdom because the human brain has differentially evolved to be highly sensitive to social norms, defined as ‘learned behavioural standards shared and enforced by a community’ (Chudek & Henrich, 2011). If the norm-psychology hypothesis is correct, *Gitanos*’ and non-*Gitanos*’ behaviour in the experiment should reflect such differences in cultural norms, which work as proximate-level behavioural explanations driven by the internalisation of the group's norms. In Text S1 we explore some of these cultural differences and the associated (proximate-level) hypotheses in more detail, in particular those related to norm enforcement institutions and gender roles. Table 1 shows the predictions for the social-benefits account, in both its most radical and comprehensive interpretations, as well as for deterrence theory.

**Table 1.** Summary of the theoretical predictions for research questions 1–3

	Predictions – condition/research question		
Account	Homogeneous Question 1	Mixed Question 2	Overall (homogeneous and mixed) Question 3
Social benefits (radical)	*I*^*G*^ < *I*^NG^	*I > O*	*I*_H_ *> I*_M_ *> O*
		----------------------	----------------------------
		*antisocial: I < O*	*antisocial: I*_H_ ≈ *I*_M_ *< O*
Social benefits (comprehensive)	*I*^G^ < *I*^NG^	*I*^G^ > *O*^G^ *I*^*NG*^ < *O*^*NG*^	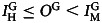 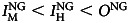
		-----------------------	----------------------------
		*antisocial: I < O* (especially *G*)	*antisocial: I*_M_ ≈ *I*_H_ *< O* (especially *G*)
Deterrence	*I*^G^ ≥ *I*^NG^	*I < O* (especially *G*)	*I*_M_ *< I*_H_ *< O* (especially *G*)

Notes: *I* = punishment targeted at ingroups; *O* = punishment targeted at outgroups. Superscripts G and NG refer to *Gitano* and non-*Gitano* punishers, while subscripts H and M refer to homogeneous groups and mixed groups, respectively. Predictions for antisocial punishment are preceded by ‘*antisocial*:’.

#### 1. Is altruistic punishment more, or less, frequent in ethnocultural groups in which individuals are more strongly related (whether owing to kinship or to closer/frequent interactions)?

Cultural group selection theorists have long argued that peer punishment, in contrast to other sanctioning mechanisms such as centralised punishment or ostracising, should be more frequently observed in larger, more complex societies with more impersonal interactions. Past cross-cultural studies support this prediction (Marlowe et al., 2008; Henrich et al., 2010). The reason is that the costs associated with punishing (e.g. damaging long-term relationships or the possibility of counter-punishment against the punisher or her family, to which we can add the direct negative impact on the punisher's inclusive fitness, see below) are higher in more tightly knit communities, whereas the effectiveness of other mechanisms such as reciprocity and reputation is relatively lower in larger societies (Henrich & Muthukrishna, 2021). In fact, within-group selection pressures might act against peer punishment in environments of high relatedness owing to the negative impact of punishment on the fitness of individuals who share genes with the punisher and, consequently, on the punisher's inclusive fitness (Gardner & West, 2004). Previous studies suggest that this might indeed be the case (Henrich & Henrich, 2014; Schulz et al., 2019). Therefore, we should find that *Gitanos* use altruistic punishment *less* than non-*Gitanos* in the experiments. Our own ethnographic data, interpreted from a norm-psychology perspective, also align nicely with this prediction insofar as the culture of individual liberty observed in the *Gitano* population (San Román, 2010; Gamella, 2011) suggests that it is not the individual's responsibility to sanction co-ethnics’ sporadic instances of non-cooperation; solidarity and forgiveness may be the intuitive reaction (Brañas-Garza et al., 2006, see Text S1). The prediction of the social-benefits account, in any of its versions, for homogeneous groups is thus *I*^G^ < *I*^NG^, where *I* stands for punishment of ingroup free-riders while superscripts G and NG refer to *Gitano* and non-*Gitano* punishers, respectively (see Table 1).

If current punishment behaviour represents the misfiring of a pan-human psychology that emerged in an ancestral past where kinship- and closeness-based interactions prevailed, as prescribed by mismatch-related theories, one might expect misfiring to be more prominent among *Gitanos*. In other words, if punishment evolved because it yielded direct or indirect inclusive fitness benefits to the punisher but is ‘mistakenly’ used in the current scenario owing to the existence of cues evoking the ancestral scenario, *Gitanos* might in general punish free-riders *more* than non-*Gitanos* owing to their higher genetic relatedness and closer daily-life relationships: socioecological conditions that are more similar to those faced by ancestral humans. Deterrence becomes indeed more necessary as the probability of repeated interaction increases. Since cultural differences are not the focus of deterrence theory, we conservatively characterise its prediction in this regard as *I*^G^ ≥ *I*^NG^ (Table 1).

#### 2. Are ingroups and outgroups punished differently for the same behaviours?

The above hypotheses about ethnic differences were developed considering each ethnocultural group separately and are therefore focused on individuals’ behaviour in homogeneous groups. Now we turn to the mixed groups.

A somewhat radical interpretation of the social-benefits account suggests that ingroup wrongdoers should be always punished more strongly than outgroup wrongdoers, as punishment behaviour is argued to play a fundamental role in maintaining ingroup cohesiveness (Henrich et al., 2010; Chudek & Henrich, 2011; Richerson et al., 2016). In parallel, given the importance of intergroup conflict and parochialism for this account (Bowles, 2006; Choi & Bowles, 2007; Henrich & Muthukrishna, 2021), participants are expected to punish outgroup (vs. ingroup) cooperators more spitefully/antisocially (Herrmann et al., 2008; Brañas-Garza et al., 2014) as harming the outgroup helps one's own cultural group outcompete other groups. Therefore, according to this radical interpretation of the social-benefits arguments, we should observe relatively more altruistic punishment of ingroup (vs. outgroup) free-riders and more antisocial punishment of outgroup (vs. ingroup) cooperators in both ethnic groups (Rusch, 2014): *I > O* for altruistic punishment and *I < O* for antisocial punishment, where *O* stands for punishment of outgroups (Table 1). This is the interpretation of the social-benefits account most commonly used by advocates of deterrence theory (Delton & Krasnow, 2017). Note that mismatch-related theories, including deterrence theory, are typically silent about the role of antisocial punishment.

Nonetheless, a more comprehensive reading of the social-benefits approach suggests that, even if cooperation seems to be a human moral universal (Curry et al., 2019), each cultural group should use sanctions to enforce those social norms which are particularly beneficial for the group (see the most recent extended synthesis in Henrich & Muthukrishna, 2021). The different historical trajectories of *Gitanos* and non-*Gitanos*, with the former traditionally being a discriminated-against minority (in Spain and elsewhere; Bauer et al., 2018; Martín et al., 2019), might thus be associated with different group-level functional needs and domain-specific cooperation (Curry et al., 2019) and hence result in peer punishment being used to enforce different social norms. The key group-beneficial norm for *Gitanos* should be to protect their strong ethnic identity and ingroup reputation, thus predicting *I*^G^ > *O*^G^ in mixed groups (Table 1). For non-*Gitanos*, a norm of generalised cooperation is expected to be crucial (‘all people should cooperate’) and enforced through peer punishment. The perceived moral superiority of non-*Gitanos* over *Gitanos* and the negative stereotype of the latter when it comes to civic cooperation (Martín & Gamella, 2005; Bauer et al., 2018) should lead to stronger punishment of *Gitanos*’ free-riding by non-*Gitanos*, as a *Gitano*'s lack of cooperation confirms the stereotype (thus it is less likely perceived as an error), hence *I*^NG^ < *O*^NG^ (Table 1). This means that a moral-superiority argument yields predictions consistent with the ingroup bias observed in many third-party punishment experiments (see above). The next question tackles this issue in greater depth. Antisocial punishment of outgroup cooperators, as in the radical interpretation, should be stronger than that of ingroup cooperators, but a comprehensive reading requires consideration of the cultural trajectories and ethnic identity strength of both groups. Thus, the *I < O* prediction for antisocial punishment is expected to be sharper among *Gitanos* (Table 1).

Applied to intergroup encounters, deterrence theory predicts that the punishment psychology is programmed to defend the interests of the punisher's allies (ingroups in this case) as this typically deters future mistreatment of oneself (Krasnow et al., 2016; Delton & Krasnow, 2017). Although this extended deterrence logic has typically been used to explain third-party punishment – while the PGP has components of both second- and third-party punishment (see Discussion) – it can be translated to our design: participants should punish outgroup free-riders the most and ingroup free-riders in the mixed groups the least because in these groups it is clear whose interests are (not) to be defended. Hence the prediction of deterrence theory would be *I < O* in mixed groups (Table 1). In contrast to the above, nonetheless, these patterns are expected to be sharper among *Gitanos*, who have stronger interests in ingroups vs. outgroups owing to both genetic and coalitional reasons.

#### 3. Do individuals punish ingroups differently when there are outgroups in the group?

A radical interpretation of the social-benefits account predicts that the punishment of ingroup free-riders should be stronger when all the interactants are ingroups as the cooperation norm is clear, leading to the overall prediction that *I*_H_ *> I*_M_ *> O*, where subscripts H and M refer to the homogeneous and mixed conditions, respectively (Table 1). The antisocial punishment of ingroup cooperators should be similarly low in both conditions, so *I*_H_ *≈ I*_M_ *< O* (Table 1).

A comprehensive reading of the social-benefits account, however, provides more nuanced predictions. As is often the case with ethnic minorities, compared with the non-*Gitano* majority, *Gitano* people display stronger group identity and higher group entitativity (i.e. the group is perceived to be a unified, single agent by outgroups and, consequently, the behaviour of the whole group is often automatically identified with the behaviour of its individual members; Hamilton et al., 1998). Previous evidence suggests that individuals from groups with higher entitativity are more prone to feelings of collective responsibility when the group identity of an ingroup wrongdoer is salient, as in our mixed condition (Kardos et al., 2019). That is, during intergroup encounters, members of a wrongdoer's group often react with feelings of shame and anger and may take actions to protect the ingroup reputation, such as sanctioning the ingroup wrongdoer, and this is stronger in groups with sharper identification (Marques et al., 1988; Lickel et al., 2007). According to this argument, we should expect that *Gitanos* display a stronger sense of collective responsibility and, therefore, punish ingroup free-riders more harshly in mixed than in homogeneous groups, as ethnic identity is made salient in the former condition. Therefore, different social norms are functional for different cultural groups: while for non-*Gitanos* (displaying the characteristics of large, impersonal societies) a norm of universal, generalised cooperation is expected to be crucial (Henrich et al., 2010), for *Gitanos* the key norm might be to protect the ingroup reputation against identity threats (Akerlof & Kranton, 2000), spurred by the group's high degree of entitativity.

The social benefits associated with ingroup reputation are therefore expected to be key for a negatively stereotyped cultural group such as the *Gitano* minority, leading to the overall prediction: 
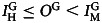
 (Table 1). That is, *Gitanos* should punish very little in homogeneous groups where the *Gitano* within-group norms of individual liberty and ingroup solidarity prevail. In the mixed condition, other norms are at play, in particular, the non-*Gitano* norm of generalised cooperation and the *Gitano* norm of defending ingroup identity and reputation, spurred by a negative stereotype. The same stereotype and the associated moral inferiority of the *Gitanos* in terms of civic cooperation should lead to more punishment of *Gitano* free-riders by non-*Gitanos*. Since the stereotype of the target interacts with observed uncooperativeness (the more negative the stereotype the less likely free-riding will be seen as an unintentional error), non-*Gitanos* should punish *Gitano* free-riders in mixed groups more harshly than non-*Gitano* free-riders in the homogeneous groups and, as punishment opportunities are limited, they should punish non-*Gitano* free-riders in mixed groups the least. This leads to similar predictions to the deterrence hypothesis below, but for non-*Gitanos* only: 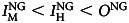 (Table 1). Regarding antisocial punishment, if a more comprehensive social-benefits lens is used, the *I*_H_ *≈ I*_M_ *< O* prediction from the radical interpretation above is expected to be sharper among *Gitanos* (Table 1).

As mentioned, an extended deterrence argument would predict that ingroup wrongdoers receive less punishment than outgroup wrongdoers in the mixed condition. Moreover, the punishment of ingroup free-riders in the homogeneous groups should fall somewhere in between. The rationale is that, in the mixed groups, ingroups are the victims of outgroups’ wrongdoing, whereas (leaving the punisher apart) only outgroups are the victims of ingroups’ wrongdoing. Therefore, it is evident that it is the ingroup's interests that should be protected through punishment. In the homogeneous groups, ingroups are both victims and perpetrators so it is less clear who should be defended. This leads to the overall prediction *I*_M_ *< I*_H_ *< O*, which should be more clearly observed among *Gitanos* (Table 1) as their interests are relatively more dependent upon the ingroups’ individual welfare.

#### 4. Does gender moderate the effect of ethnicity or group-level ethnic composition on punishment?

The fact that males tend to gain leadership relative to females in intergroup encounters does not seem to be contested, regardless of whether the focus is on cultural evolution and group selection or on genetic evolution and individual/sexual selection (Mathew & Boyd, 2011; McDonald et al., 2012; Micheletti et al., 2018). We may accordingly expect to observe that females punish comparatively less in the mixed than in the homogeneous condition relative to males. Building upon the ethnographic evidence mentioned earlier, however, the norm-psychology approach inherent to social-benefits theories suggests that *Gitano* females might punish less than *Gitano* males (and less than non-*Gitano* males and females as well) in both experimental conditions because males are always present in the interacting groups. Under these circumstances, *Gitano* norms indicate that females should let males lead the public interaction and thus probably the responsibility to punish non-cooperators. Deterrence theory, on the other hand, does not provide predictions for the existence of culture-specific gender differences.

## Methods

**Figure 1.**
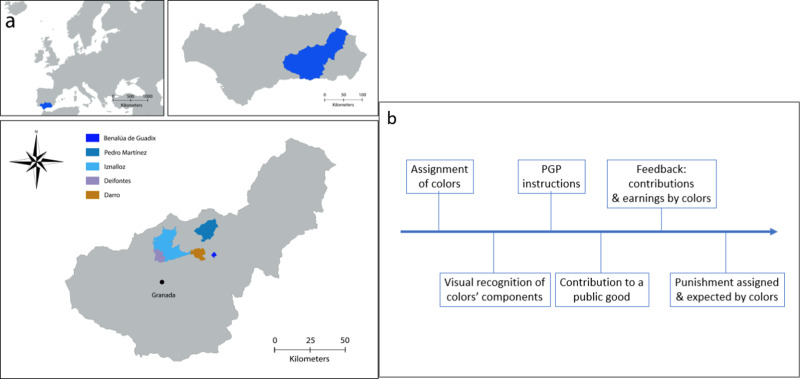
(a) Five semi-rural towns in southern Spain (Granada, Andalusia): Benalúa de Guadix, Darro, Deifontes, Iznalloz, and Pedro Martínez. (b) Structure of the experiment.

Five semi-rural towns in southern Spain (Granada, Andalusia) with comparable demographic characteristics hosted our experiments: Benalúa de Guadix, Darro, Deifontes, Iznalloz, and Pedro Martínez (see Figure 1a). As a call for participation, a €5 show-up fee and a drink and *tapa* at the end of the experiment were offered. Recruitment of non-*Gitano* participants was mainly done through the town halls (the activity was publicly announced as a study for the University of Granada and individuals informed the staff about their interest in participating, although some people just showed up to the experiment and were able to participate if there were available slots). The town halls, however, did not provide such a good means to contact *Gitanos* since they are typically less involved in towns’ official collective activities, so we needed to encourage the participation of *Gitanos* using other methods. Although we asked the town halls staff to advertise the event among *Gitano* families, we also relied on our fieldwork knowledge of *Gitano* families to recruit local members.

Two of the main researchers (AME and JFG) announced the study in several *Gitano* households from different family lines and asked our acquaintances there to bring their relatives and friends (‘*su gente*’, ‘their folks’) to the experiments. While it is true that this makes a difference in the recruitment method for *Gitano* and non-*Gitano* participants, it is important to note that (a) being unable to fill the sessions with *Gitano* participants was the main risk to be avoided, (b) many of the non-*Gitano* participants also ‘brought some of their folks’ to the experiment, (c) the same process was used for all the experimental sessions (see below) so that any treatment condition effect on behaviour cannot be attributed to differences in the recruiting method, (d) owing to our group's long relationship with the *Gitanos* in this area, the people contacted by the researchers covered a fairly representative share of the *Gitano* population in each town, (e) there is a small number of *Gitano* family lines in each town owing to the high relatedness of all the *Gitano* inhabitants, thus, ‘their folks’ were not simply their close family unit but typically included their extended family as well (potentially also friends), and these families tend to be very large, and (f) the system used to assign colours to people (those coming together tended to receive scarves of the same colour; see below) minimised the probability that two folks interacted in the experiment because only one person per colour was assigned to each PGP group. In sum, given these features that reduced the impact of the recruitment method, we consider that it did not dramatically influence the results. Yet self-selection and non-representativeness can still be an issue, as in most lab and field experiments (see Exadaktylos et al., 2013; for a thorough discussion).

In each location, we ran two experimental sessions in a between-subjects design: one ethnically homogeneous session (either all *Gitanos*, in two locations, or all non-*Gitanos*, in three locations) and one ethnically mixed session (same number of *Gitanos* and non-*Gitanos*; one session in each of the five locations) where ethnic identity was made salient. We ensured that subjects in one session did not learn the ethnic composition of the other session prior to participating. In each of the 10 sessions, 32 participants played the one-shot PGP in eight independent groups of four people. The participants were initially evenly assigned one out of four colours using visible coloured scarves. Colours were assigned similarly in both sessions, which induced colour assignment to be dependent on ethnicity in the mixed sessions since two of the colours were assigned to *Gitanos* and the other two colours to non-*Gitanos*. This procedure was unknown to the participants and was done by giving scarves of identical colour to participants who showed up together. Since *Gitanos* and non-*Gitanos* always arrived separately, the resulting assignment of colours to ethnic groups was nearly perfect (see below).

In the mixed sessions, we subtly induced the participants to realise the link between colours and ethnicities prior to playing the game (in the homogenous sessions we made the composition of colours public as well in order to allow for comparability between conditions): the eight participants of each colour were placed together wearing their scarves and photographed by an assistant in front of the other participants. This feature of the design allowed the participants to associate cooperation decisions to ethnicities (i.e. colours) and condition their punishment decisions upon the ethnicity of the target in mixed groups. Data from post-experimental interviews indicate that most participants were able to associate ethnicities to scarf colours in the mixed sessions (even if socially desirable responding might have reduced their willingness to acknowledge this). See Figure 1b for a representation of the structure of the experiment.

For the statistical analyses, we excluded seven participants: two *Gitanos* because they participated in a homogeneous non-*Gitano* session (we learned their ethnicity ex-post) and five individuals from four different mixed sessions because their ethnicity did not match their scarf colour (including them does not qualitatively affect the results). The final sample consisted of 143 *Gitanos* and 170 non-*Gitanos*.

The basic elements of the PGP design have been reported elsewhere (Espín et al., 2012). Each four-person PGP group was composed of one randomly selected person from each (scarf) colour. Beyond colours, group membership was unknown. After deciding how much to contribute to a public good from an endowment of €10 (marginal per capita return = 0.5; thus each contributed euro cost the individual 50 cents but increased the earnings of each of the other three group members by 50 cents), the participants received feedback on their group partners’ contributions and earnings in a colour-based fashion and could then anonymously reduce other group members’ payoffs at a personal cost (cost-to-impact ratio of punishment = 1:3). Finally, the participants were also asked to state the level of punishment they expected from each group partner (no monetary incentives were used for the expectations task). Figures S1 and S2 display the contribution and punishment decision cards, respectively. Several examples of all stages of the PGP were displayed on a whiteboard to facilitate understanding of the game rules. The instructions were explained by the same researcher (PBG) in all the sessions.

After the PGP, the participants completed an unrelated task. At the end of the experiment, they were privately asked to answer a set of socio-demographic questions and received their payment. Mean earnings from the PGP were €13.34 ± 4.08 (SD).

### Statistical analysis

All statistical analyses were conducted using Stata v. 13 (Stata Corp). We implemented ordinary least squares regressions for the analysis of contributions to the public good and multilevel generalised linear mixed model regressions for the punishment decisions (i.e. the amount reduced through punishment) with random effects on the PGP group, the decision-maker and the target individual to account for the interdependence of data at these three levels. All of the regression results are reported in Tables S1–S3. In the main text, we report the coefficient, standard error (SE) and two-tailed *p*-value for each contrast obtained from regressions in columns 1a–5a. The reported standard errors are always robust to heteroscedasticity. The main (binary) explanatory variables in the regressions are the decision-maker's ethnicity (*Gitano* vs. non-*Gitano*), the experimental condition (mixed vs. homogeneous), and the decision-maker's gender (male vs. female), as well as their interactions. For the analysis of punishment behaviour in the mixed groups, we also included the target's ethnicity (*Gitano* vs. non-*Gitano*) among the main explanatory variables. Secondary explanatory variables included the difference between the decision-maker's and the target's contributions to the public good (i.e. punisher's minus target's) and the mean contribution of the other two group members. All regressions are repeated, in adjacent columns (1b–5b), with controls for the decision-maker's age (ranging from 16 to 82; mean for *Gitanos* = 34.56 ± 13.60 SD; mean for non-*Gitanos* = 49.97 ± 18.97 SD; the difference is significant, *p* < 0.01, *t*-test) and household income (ranging from to 0 to 9, corresponding to ‘0 euros/month’ and ‘more than 5,000 euros/month’ bins, respectively; mean for *Gitanos* = 1.944 ± 1.211 SD; mean for non-*Gitanos* = 3.195 ± 1.564 SD; the difference is significant, *p* < 0.01, Mann–Whitney test) as potential confounding factors (Martín et al., 2019).

### Ethics statement

All participants provided consent prior to participation. Oral informed consent was obtained because literacy was not a requirement to participate owing to the (expected) low educational level of many participants; only being able to read and write numbers was required to participate. All procedures contributing to this work comply with the ethical standards of the relevant national and institutional committees on human experimentation and with the Helsinki Declaration of 1975, as revised in 2008. Participants were treated anonymously by assigning them a numerical code in accordance with Spanish Law 15/1999 on Personal Data Protection. No association was made between their real names and the results. This procedure was checked and approved by the Vice-Dean of Research at the School of Economics of the University of Granada.

## Results

### Contributions to the public good

**Figure 2.**
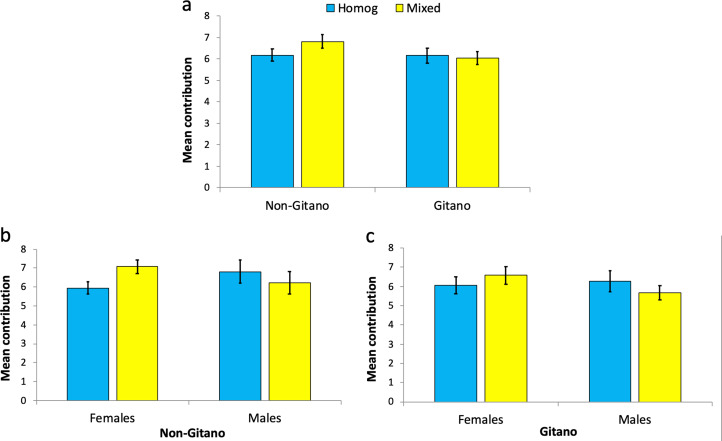
Mean contributions in homogeneous and mixed conditions. (a) Displays the data broken down by ethnicity. (b and c) Display the data broken down by gender for non-*Gitanos* and *Gitanos*, respectively. Error bars represent standard error of the mean.

The results on participants’ cooperation, as measured by their contributions to the public good, are displayed in Figure 2. No main effect of ethnicity (coefficient of *gitano* = −0.361 ± 0.321 SE, *p* = 0.26; Table S1, column 1a) or condition (coefficient of *mixed* = 0.306 ± 0.312 SE, *p* = 0.33; Table S1, column 1a) on contributions was found. The interaction between these two variables was not significant either (coefficient of *gitano* × *mixed* = −0.733 ± 0.620 SE, *p* = 0.24; Table S1, column 2a) and all possible comparisons report *p* > 0.10 according to joint-significance Wald tests on the model estimates. Adding controls for age and household income does not qualitatively change the results (Table S1, columns 1b and 2b). Therefore, contributions did not differ between ethnic groups (in aggregate or within each condition) or between conditions (in aggregate or within each ethnic group). Contribution levels were relatively high (well above 60% of the endowment on average; see Ledyard, 1995). Given that the threat of punishment introduces incentives to cooperate strategically and therefore contributions do not necessarily reflect a ‘pure’ preference for cooperation, the finding of similar average contribution levels across cultural groups and conditions could be due to multiple factors. Note that, owing to this multiplicity of motives, we did not put forward any hypotheses about the groups’ cooperation levels and all of the analyses on contribution behaviour are thus exploratory.

However, we observed a significant interaction between gender and condition (coefficient of *mixed* × *male* = −1.543 ± 0.641 SE, *p* = 0.02; Table S1, column 4a; see Figure 2b and c). Across both cultural groups (apparently more clearly among non-*Gitanos* although the three-way interaction ethnicity × condition × gender was not significant, coefficient = 0.613 ± 1.336 SE, *p* = 0.65; Table S1, column 5a), we found that females contributed more in the mixed than in the homogenous groups (coefficient of *mixed* = 0.921 ± 0.384 SE, *p* = 0.02; Wald test on Table S1, column 4a), while the opposite was observed for males (although not significantly so; coefficient of *mixed* = −0.622 ± 0.515 SE, *p* = 0.23; Wald test on Table S1, column 4a). As a result, males cooperated significantly less than females in the mixed groups (coefficient of *male* = −0.938 ± 0.435 SE, *p* = 0.03; Wald test on Table S1, column 4a), but similarly in the homogeneous groups (coefficient of *male* = 0.605 ± 0.486 SE, *p* = 0.21; Wald test on Table S1, column 4a). Again, controlling for age and household income does not qualitatively affect the results (Table S1, columns 4b and 5b).

### Aggregate punishment levels

**Figure 3.**
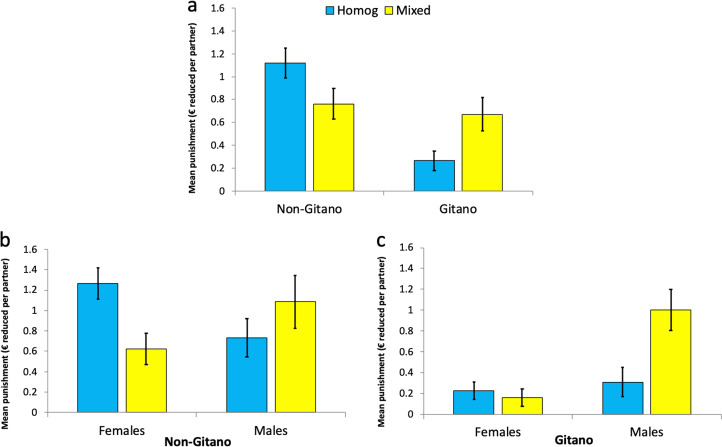
Mean aggregate punishment in homogeneous and mixed conditions. (a) Displays the data broken down by ethnicity. (b) (non-*Gitanos*) and (c) (*Gitanos*) display the data broken down by ethnicity and gender. Error bars represent robust standard error of the mean clustered at the PGP group level.

Figure 3 summarises the results regarding punishment behaviour. We observed a significant main effect of ethnicity, indicating that, in general, *Gitanos* punished less than non-*Gitanos* (coefficient of *gitano* = −0.362 ± 0.116 SE, *p* < 0.01; Table S2, column 1a). The treatment condition did not yield a significant estimate (coefficient of *mixed* = −0.065 ± 0.148 SE, *p* = 0.66; Table S2, column 1a). A significant ethnicity × condition interaction (coefficient of *gitano* × *mixed* = 0.807 ± 0.228 SE, *p* < 0.01; Table S2, column 2a) reveals that *Gitanos* punished much less than their non-*Gitano* counterparts in the homogeneous groups (coefficient of *gitano* = −0.870 ± 0.156 SE, *p* < 0.01; Wald test on Table S2, column 2a), but there were no ethnic differences in the mixed groups (coefficient of *gitano* = −0.063 ± 0.157 SE, *p* = 0.69; Wald test on Table S2, column 2a; see Figure 3a). The intergroup encounter triggered by the mixed condition thus exerted substantial and differential effects on both sides: *Gitanos* increased their punishment level (coefficient of *mixed* = 0.389 ± 0.168 SE, *p* = 0.02; Wald test on Table S2, column 2a) while non-*Gitanos* reduced it (coefficient of *mixed* = −0.418 ± 0.193 SE, *p* = 0.03; Wald test on Table S2, column 2a), as compared with the homogenous condition.

There was also a significant interaction between ethnicity and gender on punishment (coefficient of *gitano* × *male* = 0.574 ± 0.231 SE, *p* = 0.01; Table S2, column 3a). This stems from a higher level of punishment implemented by males compared with females among *Gitanos* (coefficient of *male* = 0.427 ± 0.130 SE, *p* < 0.01; Wald test on Table S2, column 3a). No aggregate gender difference in punishment was found among non-*Gitanos* (coefficient of *male* = −0.147 ± 0.189 SE, *p* = 0.44; Wald test on Table S2, column 3a).

Finally, a significant interaction was also found between condition and gender (coefficient of *mixed* × *male* = 1.084 ± 0.223 SE, *p* < 0.01; Table S2, column 4a). Specifically, we observed a higher level of punishment by males (coefficient of *mixed* = 0.593 ± 0.198 SE, *p* < 0.01; Wald test on Table S2, column 4a) and a lower level of punishment by females (coefficient of *mixed* = −0.492 ± 0.158 SE, *p* < 0.01; Wald test on Table S2, column 4a) in the mixed than the homogenous groups (see Figure 3b and c). This results in males punishing less than females in the homogenous groups (coefficient of *male* = −0.373 ± 0.136 SE, *p* < 0.01; Wald test on Table S2, column 4a), but more than females in the mixed groups (coefficient of *male* = 0.712 ± 0.174 SE, *p* < 0.01; Wald test on Table S2, column 4a). Although the three-way interaction ethnicity × condition × gender was not significant (coefficient = −0.476 ± 0.434 SE, *p* = 0.27; Wald test on Table S2, column 5a), it can be seen that *Gitano* females almost never used punishment in either condition. In other words, punishment by *Gitano* females was nearly nonexistent regardless of the condition whereas the level of punishment implemented by *Gitano* males, which was negligible in the homogeneous groups, turned out to be rather high in the mixed groups. Among non-*Gitanos*, females punished less while males punished more in the mixed than in the homogeneous groups. As before, adding controls for age and household income does not alter any of the above findings (Table S2, columns 1b–5b).

### Altruistic and antisocial punishment

**Figure 4.**
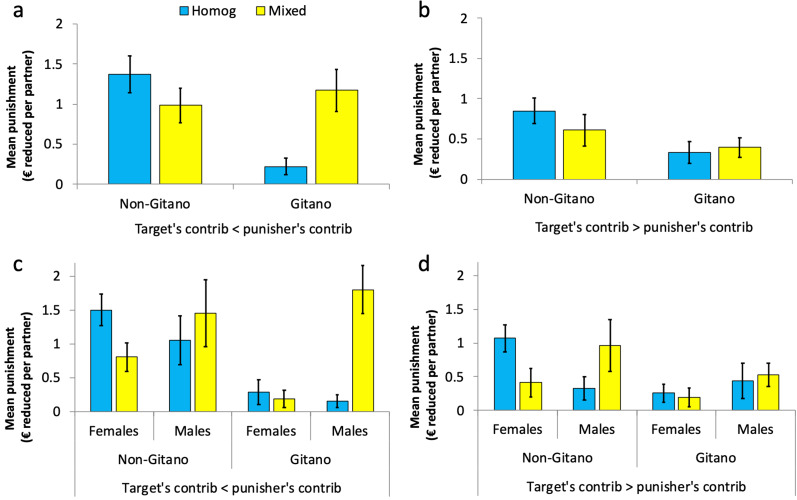
Mean altruistic and antisocial punishment in homogeneous and mixed conditions. (a) (Altruistic punishment) and (b) (antisocial punishment) display the data broken down by punisher's ethnicity. (c) (Altruistic punishment) and (d) (antisocial punishment) display the data broken down by punisher's ethnicity and gender. Error bars represent robust standard error of the mean clustered at the PGP group level.

In all the above regressions, the higher the difference between the punisher's contribution and the target's contribution (punisher's minus target's), the stronger the punishment (in all cases, coefficient of *differ* > 0.07, SE < 0.02, *p* < 0.01; Table S2, columns 1a–5a), thus indicating that more intense free-riding receives firmer punishment, as is standard in the literature (Fehr & Gächter, 2002; Herrmann et al., 2008; Espín et al., 2012). However, we also observe some instances of spiteful, antisocial punishment targeted at cooperators. When disentangling between ‘altruistic’ punishment (the target contributed less than the punisher) and ‘antisocial’ punishment (the target contributed more than the punisher) in panels (a) and (b) of Figure 4, we see that the rather strong punishment implemented by *Gitanos*, in particular males (panels (c) and (d) break down the data by gender), in the mixed compared with the homogeneous groups is due almost uniquely to altruistic punishment since their level of antisocial punishment was still very low in the mixed groups. The remaining results mentioned above do not appear to crucially depend, at least qualitatively, on whether punishment is altruistic or antisocial.

### Ethnocultural identities and punishment in mixed groups

**Figure 5.**
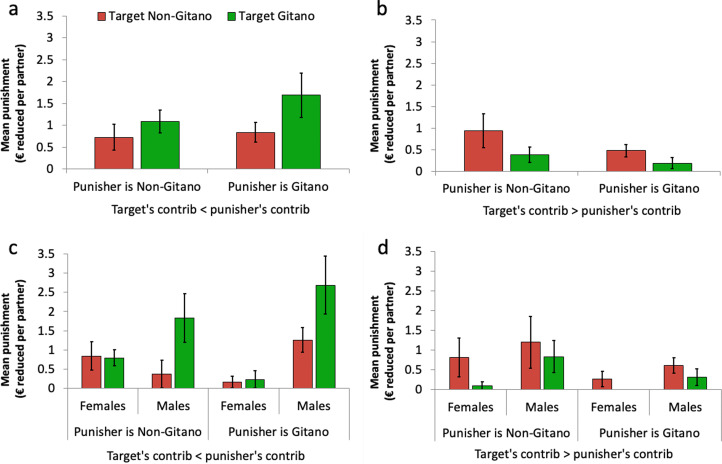
Mean punishment on *Gitano* and non-*Gitano* targets in mixed groups. (a) (Altruistic punishment) and (b) (antisocial punishment) display the data broken down by punisher's ethnicity. (c) (Altruistic punishment) and (d) (antisocial punishment) display the data broken down by punisher's ethnicity and gender. Error bars represent robust standard error of the mean clustered at the PGP group level.

It remains to be determined whether participants punished differently in the mixed groups depending on the cultural identity of the target (recall that the punisher knew the target's ethnicity but not her personal identity). In Figure 5, we display the mean punishment levels imposed on *Gitano* and non-*Gitano* targets in the mixed groups. We find that, *regardless of the punisher's ethnicity*, *Gitano* targets received less antisocial punishment and more altruistic punishment than non-*Gitano* targets for the same behaviours (significant interaction between the target's ethnicity and contribution difference: coefficient of *targetgit* × *differ* = 0.105 ± 0.037 SE, *p* < 0.01; Table S3, column 4a; the three-way interaction with punisher's ethnicity was not significant: coefficient = 0.057 ± 0.074 SE, *p* = 0.45; Table S3, column 5a; see Figure 5a and b). *Gitano* targets got punished significantly less than non-*Gitano* targets when they cooperated more than the punisher (coefficient of *targetgit* between −0.323 ± 0.162 SE and −0.954 ± 0.347 SE, *p* < 0.05, for differences between €4 and €10; Wald test on Table S3, column 4a), whereas *Gitano* targets got punished more than non-*Gitano* ones when they cooperated less than the punisher (coefficient of *targetgit* between 0.414 ± 0.213 SE and 1.151 ± 0.439 SE, *p* < 0.05 for differences between €3 and €10; Wald test on Table S3, column 4a). Still, note that antisocial punishment was much less frequent than altruistic punishment. As can be seen in Figure 5c and d, the difference in altruistic punishment between *Gitano* and non-*Gitano* targets is due solely to male punishers, whereas the difference in antisocial punishment is similar across genders, although it appears to be stronger among non-*Gitano* female punishers. All of these results also remain after controlling for age and household income (Table S3, columns 1b–5b).

To summarise, in contrast to the high punishment levels observed among non-*Gitanos*, *Gitanos* practically did not punish the uncooperativeness of other *Gitanos* in the homogeneous groups but (in particular males) severely punished such behaviour in the mixed groups with non-*Gitanos*. Non-*Gitano* males, on the other hand, also retaliated more harshly against *Gitano* free-riders than against non-*Gitano* ones in the mixed groups. Regarding the antisocial punishment of cooperators, the results are somehow weaker: while participants, regardless of their ethnicity, tended to target more punishment at non-*Gitano* than *Gitano* cooperators in the mixed groups, the levels of antisocial punishment were relatively low (especially compared with those of altruistic punishment).

### A closer look into the basic competing hypotheses

**Figure 6.**
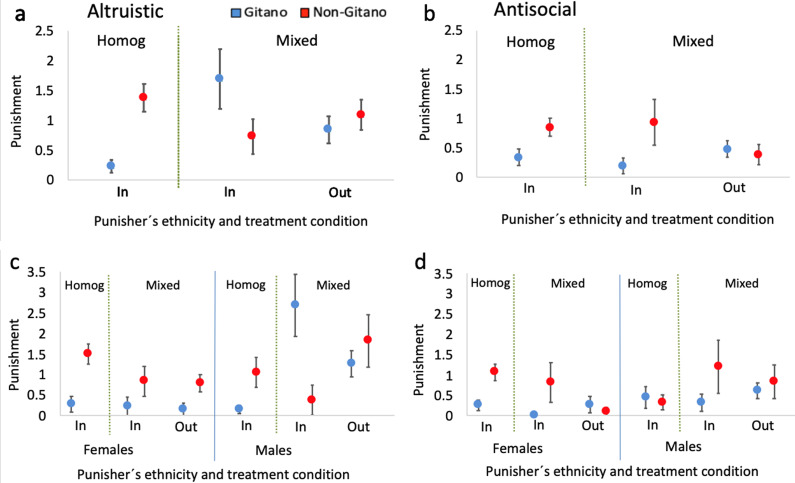
Mean altruistic and antisocial punishment targeted at ingroups and outgroups. (a) (Altruistic punishment) and (b) (antisocial punishment) display the data broken down by punisher's ethnicity and treatment condition (ingroup-homogeneous, ingroup-mixed and outgroup-mixed). (c) (Altruistic punishment) and (d) (antisocial punishment) display the data broken down by punisher's ethnicity, treatment condition and punisher's gender. Error bars represent robust standard error of the mean clustered at the PGP group level.

In Figure 6a and b we rearrange the above results regarding altruistic and antisocial punishment in a manner that facilitates comparison with the testable predictions outlined in Table 1.

Our research question 1 has a clear answer: with regards to altruistic punishment (Figure 6a), from the homogeneous condition we observe that *I*^G^ < *I*^NG^ (*p* < 0.01)_._ Thus, the punishment targeted at ingroup free-riders in the homogeneous groups is higher among non-*Gitanos* than among *Gitanos*, as predicted by both versions of the social-benefits hypothesis.

Our research question 2 has a more complex answer, however. In the mixed groups, we can see that *I*^G^ > *O*^G^ and *I*^NG^ < *O*^NG^ hold for altruistic punishment (Figure 6a; *p* < 0.05 for differences between the punisher's and the target's contributions larger than €3 in both cases; see above). Here, the results for *Gitano* punishers match both the radical and comprehensive social-benefits interpretations but not the deterrence one, while the behaviour of non-*Gitano* punishers is consistent with both the comprehensive social-benefits and the deterrence predictions (with the caveat that deterrence theory predicts that these patterns should be especially evident among *Gitano* punishers). With regards to the antisocial punishment of cooperators in the mixed groups (Figure 6b), for which deterrence theory does not pose any clear prediction, we observe *I*^G^ < *O*^G^ and *I*^NG^ > *O*^NG^ (*p* < 0.05 for differences between the punisher's and the target's contributions larger than €4 in both cases; see above). Thus, the social-benefits prediction holds among *Gitano* punishers, which aligns well with a comprehensive interpretation that these patterns should be sharper for *Gitanos*. The result for non-*Gitanos*’ antisocial punishment, however, does not seem to match the predictions of any of the proposed accounts.

The answer to our research question 3 is also intricate. Regarding altruistic punishment (Figure 6a), we observe that 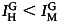 and 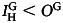 hold among *Gitanos* (both *p* < 0.01 for all possible differences between the punisher's and the target's contributions), which added to the results from question 2 leads to 
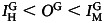
, thus being consistent with the comprehensive social-benefits account. For non-*Gitano* punishers, however, we observe 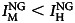 (*p* < 0.10 for differences between the punisher's and the target's contributions larger than €1) and 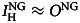 (*p* > 0.20 for all contribution differences), which combine with the results from question 2 into 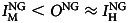. Non-*Gitano* punishers behave to a considerable extent consistent with the predictions of both the comprehensive social-benefits and deterrence theories, especially considering the behaviour of males (note that our sample has a greater proportion of females than males, 59% vs. 41%) for whom a sharp 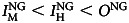 is observed (see below; Figure 6c). Nonetheless, the deterrence approach would predict these patterns to be more evident among *Gitanos* than non-*Gitanos*. Regarding antisocial punishment (Figure 6b), we observe 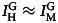 and 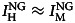 (both *p* > 0.10 for all contribution differences), which added to the results from question 2 lead to 
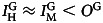
 and 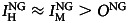. The results on antisocial punishment for *Gitanos*, but not for non-*Gitanos*, thus match the predictions of the social-benefits account, especially considering the comprehensive interpretation that led us to expect a higher prevalence of antisocial punishment of outgroup vs. ingroup cooperators among *Gitano* punishers.

Our research question 4 tackles gender differences. Figure 6c displays the results on altruistic punishment for male and female punishers separately. As mentioned above, *Gitano* females practically did not punish in any condition. In addition, the *I*^G^ < *I*^NG^ finding from the homogeneous groups and the 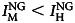 finding for non-*Gitanos* hold qualitatively regardless of the punisher's gender. Three results, however, are only driven by male punishers: 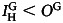 and 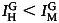 for *Gitanos* and 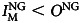 for non-*Gitanos* (as mentioned, leading to 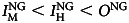). Breaking down the results on antisocial punishment by gender in Figure 6d, we see that the 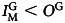 finding for *Gitanos* and the 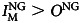 observed for non-*Gitanos* hold qualitatively for both males and females (with the disclaimer that *Gitano* females punish very little). In general, the gender differences are largely consistent with the ‘male warrior’ hypothesis, stating that males should punish comparatively more in the mixed vs. homogeneous groups relative to females. The norm-psychology prediction based on our ethnographic record that *Gitano* females should barely punish in either condition owing to the presence of males is also supported by the data.

## Discussion

We find that non-*Gitanos* punish more than *Gitanos* in homogeneous groups. This is consistent with the prediction of the social-benefits account that peer punishment should be primarily used in large-scale societies in which many sporadic interactions take place among unrelated individuals and hence other mechanisms such as kin selection and reciprocity are less effective. The results of Henrich and Henrich (2014) suggest that relatedness might reduce the willingness to punish others, since they found that individuals more genetically related to the average member of the ‘yavusa’ in a Yasawan sample (Fiji Islands) tended to punish less as third-party observers. Moreover, in such a highly genetically related population, punishment was comparatively infrequent, and zero offers were very often accepted in both ultimatum and third-party punishment games, whereas actual offers were on average quite high (i.e. ‘fair’). This matches the ultimatum game results of Brañas-Garza et al. (2006) with a sample of Spanish *Gitanos* in Madrid, where high offers were observed even though much lower offers would have gone unpunished. In cultural groups organised around tight kinship-based networks, peer punishment may not be favoured to enforce daily-life group cooperation if other mechanisms such as ostracism or collective/centralised punishment institutions represent lower-cost sanctioning solutions (Henrich et al., 2010; Mathew, 2017; Henrich & Muthukrishna, 2021). Past experimental research also suggests that cooperation, but *not* punishment, increases with cues of kin density in PGP groups (Krupp et al., 2008). The exact role of genetic relatedness (between the punisher, the victim(s), and the wrongdoer) for punishment behaviour is yet to be systematically assessed, however; future research should provide such a systematic evaluation.

*Gitanos*, who have a strong sense of ethnic identity and are negatively stereotyped regarding civic cooperation, targeted punishment mainly at *Gitano* wrongdoers when they interacted with non-*Gitanos* in the mixed groups but did not punish ingroup free-riders in the only-*Gitano* homogeneous groups. Non-*Gitanos*, for whom a norm of universal cooperation is expected to be key, punished ingroup free-riders less than (negatively stereotyped) outgroup free-riders in mixed groups. The results for both *Gitanos* and non-*Gitanos* are consistent with the comprehensive social-benefits interpretation that punishment may be modulated by between-group vs. within-group encounters in a culture-specific way, while the results for non-*Gitanos* match the predictions of deterrence theory.

At the proximate level, *Gitanos* seem to use punishment only in response to a clear threat to group identity (Akerlof & Kranton, 2000): that of being seen as less cooperative than non-*Gitanos*. The negative emotions triggering punishment (Fehr & Gächter, 2002; Crockett et al., 2013) among *Gitanos* would thus emanate from the possibility of comparison between the two ethnic groups. Previous research indicates that, during intergroup contact, feelings of identity threat and collective responsibility are particularly likely to be aroused among individuals with a stronger group identification (Marques et al., 1988; Hamilton et al., 1998; Dovidio et al., 2008; Kardos et al., 2019). It can thus be inferred that the key norm for *Gitanos* (that which is to be enforced through punishment) is not cooperation *per se*, but preserving an ethnic identity of which they are proud. In fact, in the homogeneous condition, a common comment by *Gitano* participants during the post-experimental interview when informally asked about their perception of punishment opportunities (i.e. ‘the possibility of reducing others’ earnings’) was that punishing others makes no sense at all. ‘Destroying others’ money and paying for it!’ (subject 25) was seen as something weird, irrational and very negative by *Gitanos* in the homogeneous condition. Comments of this type were nonexistent in the mixed condition (as well as in the only-non-*Gitano* homogeneous condition), as if the reasons for punishing others were evident for everyone. In fact, even though the beliefs elicitation was not incentivised and should therefore be taken with caution, participants’ expectations seem to match their behaviour to a large extent: *Gitanos* expected much less punishment than non-*Gitanos* in the homogeneous groups (*p* < 0.01; same regression specification as for punishment decisions) and expected more punishment in the mixed than in the homogeneous groups (*p* = 0.05; Wald test).

To a large extent, our results for *Gitanos* in the mixed groups are coherent with previous findings from ultimatum game experiments (McLeish & Oxoby, 2007, 2011; Mendoza et al., 2014) and multilateral gift-giving (non-standard) third-party punishment games (Shinada et al., 2004) using identity manipulations, which have found that ingroup wrongdoers are more strongly punished than outgroup wrongdoers. However, this seems at odds with most results from standard third-party punishment experiments in which harsher punishment has been observed when the victim is an ingroup of the third party (i.e. the punisher) and the norm violator is an outgroup, compared with other combinations (Bernhard et al., 2006; Goette et al., 2006; Jordan et al., 2014; Schiller et al., 2014; Delton & Krasnow, 2017; but see Shinada et al., 2004 for a non-standard design with different results). In contrast to results with adults, a recent third-party punishment experiment found that 3- to 4-year-olds, but not older children (see also Jordan et al., 2014), inflict harsher punishment on ingroup than outgroup norm-violators (Yudkin et al., 2019). Yet there are differences between the multilateral cooperation environment of our PGP and the framework posed by the third-party punishment game in those experiments. Importantly, punishers might have been more cooperative than the target, or less, in the third-party punishment game. However, this fundamental detail – which informs about the true (altruistic vs. antisocial) nature of punishment (Herrmann et al., 2008; Brañas-Garza et al., 2014; Espín et al., 2015) – is by design unknown (but see Shinada et al., 2004), in contrast to the PGP. One recent PGP experiment with ethnic minorities in China also found that ingroup free-riders are punished less, and less than outgroup free-riders, when there are outgroups in the group; yet, the game is not one-shot but repeated, thus other motives might be at play (Mantilla et al., 2021).

Non-*Gitanos*’ sanctioning behaviour in mixed groups, on the other hand, seems closer to those previous findings: they punish outgroup wrongdoers harshly but not ingroup ones. Therefore, their behaviour seems inconsistent with the radical interpretation of the social-benefits account and needs a more comprehensive lens to be rationalised (see below). These patterns are instead largely coherent with the predictions of deterrence theory (Krasnow et al., 2016; Delton & Krasnow, 2017). Yet while such an approach suggests that these patterns should be more evident among *Gitanos* than non-*Gitanos* owing to the stronger genetic and coalitional links with ingroups, we observe the opposite.

The fact that ethnic minorities, and Romani groups in particular, are often perceived as if not following the collective action norms of the majority (Weyrauch, 2001; Martín & Gamella, 2005; Marushiakova & Popov, 2007; Bauer et al., 2018) and as failing to abide the majority's enforcement institutions (Gay Blasco, 1999; San Román, 2010), can explain the strong punishment of *Gitano* wrongdoers by non-*Gitano* males. This result is probably symptomatic of a sense of moral superiority (Brewer, 1999) or pretended assimilation (Dovidio et al., 2008). Previous evidence indicates that members of majority status groups are typically more concerned with not being perceived as prejudiced by the minority, whereas members of minority groups are concerned with becoming the target of the majority's prejudice (Shelton, 2003; Tropp & Pettigrew, 2005). Since the stereotype is that Romani people do not contribute to the commons and display low compliance with the majority collective action norms (Bauer et al., 2018), following those arguments, it might be natural that both non-*Gitanos* and *Gitanos*, although for different reasons, punish acts that confirm the stereotype (i.e. *Gitanos* free-riding) more firmly than acts that contradict it (i.e. non-*Gitanos* free-riding or *Gitanos* cooperating). For cultural groups in which a norm of universal cooperation is expected to be enforced through peer punishment, represented by the non-*Gitanos* in our experiments, an evolved perception of outgroups as being less moral than ingroups (which is ubiquitous and perfectly consistent with CGS theories) might lead to punish outgroup wrongdoers more than ingroup wrongdoers just for the fact that outgroups’ uncooperativeness is more likely to be perceived as intentional. Ultimately, the current results indicate that a deterrence function of punishment can be overstated if experiments are conducted with specific populations or without comparing cultural groups of different status.

In addition, we find some indication that *Gitanos* spitefully punished non-*Gitano* cooperators more than *Gitano* ones (i.e. more antisocial punishment targeted at outgroups than ingroups). This result is in line with the parochialism prediction of social-benefits theories as well (Choi & Bowles, 2007), but the level of antisocial punishment in the mixed groups was perhaps too low to draw any firm conclusion.

An important aspect uncovered by our experiments relates to the impact of gender roles within as well as across cultural groups. While females contribute more in mixed than homogeneous groups, the opposite is observed for males. Also, in contrast to what we see among females, males punish generally more in mixed than homogeneous groups (consistent with our hypothesis based on a ‘male-warrior’ account; Mathew & Boyd, 2011; McDonald et al., 2012). These two results hold similarly for both *Gitano* and non-*Gitano* participants, thus suggesting the existence of gender differences common to both cultural groups. One candidate proximate force underlying such gender differences in contributions and punishment is risk aversion (recall that we did not have predictions about contributions but only about punishment). If mixed groups are perceived as risky environments owing to the presence of outgroups, probably the safest strategy is to avoid conflict by cooperating and not punishing others. Since there is abundant evidence that, at least in patriarchal societies, females are more risk averse than males (Charness & Gneezy, 2012; with evidence suggesting a biologically informed explanation – Brañas-Garza et al., 2018), this might explain why they tend to use such a strategy to a larger extent than males. Evolutionarily, intergroup conflicts might have shaped sex differences in social behaviour through several channels, including sex differences in the demography of warfare (Micheletti et al., 2020).

However, while non-*Gitano* females’ punishment was strongly modulated by group type – high in the homogeneous and low in the mixed groups – *Gitano* females practically did not punish in either condition. This result reflects culture-specific differential gender roles for norm enforcement and is consistent with the ethnographic evidence reviewed in Text S1 suggesting that the *Gitano* cultural norms prescribe women to reduce their assertiveness in the presence of (*Gitano*) males, who should ostensibly lead social interactions in such situations. These marked gender roles are far less prevalent in the majority population. Similarly, Mantilla et al. (2021) also find females punishing generally less in repeated PGP experiments among Chinese ethnic minorities. Thus, our result also aligns well with a social-benefits approach hinging upon norm internalisation.

In sum, our results are consistent with a comprehensive interpretation of the social-benefits account for our four research questions. This conclusion does not preclude the importance of punishment as a mechanism for deterrence or that punishers’ fitness might be positively affected in some way by relative-standing or reputation gains, for example (Raihani & Bshary, 2019; Akdeniz & van Veelen, 2021), but our data indicate that cultural evolution and group selection processes need to be accounted for to explain punishment behaviour. However, several findings (in particular, those related to non-*Gitano* punishers in the mixed groups) challenge a radical view of how such processes should translate into behavioural outcomes. These findings in fact raise a number of new questions that deserve further exploration and can help qualify the predictions and interpretations of the social-benefits account.

## Data Availability

The dataset and code (STATA) are available at: http://hdl.handle.net/10481/76057

## Supplementary material

For supplementary material accompanying this paper visit https://doi.org/10.1017/ehs.2022.32. click here to view supplementary material
