# Supplementary material for: Culture and group-functional punishment behaviour
Source: Evol Hum Sci. 2022 Aug 1;4:e35. doi: 10.1017/ehs.2022.32 (PMC10426100; doi:10.1017/ehs.2022.32)
Supplement: Supplementary file 1 [file S2513843X22000329sup001.pdf]

Supplementary Materials

for

Culture and group-functional punishment behaviour

Text S1. A short overview on Spanish *Gitanos* and norm-psychology hypotheses

The *Gitanos* or *Calé*[1] are an ethnocultural minority that lives today in all Spanish regions. They are related to other Romani groups in Europe and America with whom they seem to share a remote origin from an "initial founder population" that moved westwards from the Indian subcontinent over one thousand years ago (Mendizabal et al. 2012). All these groups, however, have adapted to the surrounding groups with whom they have lived and today show some traits of familial resemblance and considerable cultural heterogeneity (Matras 2015; Piasere 2004; Fraser 1992). Even those who preserve articulated dialects of Romani language (Matras 2002) are bilingual, and thus bicultural. The *Gitanos* come from the first Romani migrations into Western Europe, which ended in the second half of the 15th century (Pym 2007; Leblon 1985). Their lifeways are the product of a long coexistence and exchange with local Spanish populations. Life in common has been marked by persecution, segregation, and discrimination, but also by cooperation and hybridization (Pym 2007; Gómez Alfaro 1998; 1999; Leblon 1985; Gamella 2011; Gamella et al. 2014b).

[1] Most Spanish Romani people call themselves *Gitanos* in both private and public settings. Minority leaders also use the term to name public institutions, such as the *Instituto de Cultura Gitana*. The first Romani groups reaching Spain in the fifteenth century were called "*Egyptanos*", as they were believed to have originated in Egypt. *Gitano* is thus synonymous with the English term "Gypsy." Many Romani activists and intellectuals reject this exonym as derogatory and prefer to be identified by their own denominations, such as Roma, Sinti, Kalé, etc. Some leaders of the growing international Roma movement and some EU authorities defend the term "Roma" for all Romani groups. We rarely heard the term "Roma" in our encounters with *Gitano* people. In Spain, *Gitanos* also refer to themselves as *Calé* (plural of *Caló*, black in Romani), but less frequently.

In this sense, *Gitanos* of Spain are often portrayed as an example of successful integration. Arguably, their treatment and living conditions are relatively favourable compared to large Romani populations living in other European societies, particularly those of Central and Eastern Europe. (For instance, George Soros, the business magnate and Roma advocate and philanthropist "called upon Spain to lead Europe in bettering the conditions of the Roma" [Peiró 2012:ix]. Similar claims have been expressed often in the international mass media.) But the rosy view of the lot of the Spanish Romani is often exaggerated and downplays the discrimination and disadvantage many *Gitano* men and women still suffer in labor, income, education, and even daily life encounters (Álvarez-Roldán et al. 2018). It is true, however, that since 1977, when the new political context brought about democracy and decentralization of the Spanish state, there have been clear improvements in their access to health care, education, and housing, but not without conflicts and rejection by local majorities.

Today, most *Gitanos* are proud of their ethnic identity, although they consider themselves autochthonous Spaniards, especially in face of the large number of foreign economic immigrants who moved to Spain in the last two decades and increased the country's ethnic and cultural diversity. *Gitanos* speak the languages and dialects of the regions where they live and have lost most of their old trades and occupations. They have, however, developed other differences in religious expression and mobilization or in gender and marriage rituals, as well as in reproductive patterns, to construct and vindicate their shared identity (Gay Blasco 1999; Cantón 2010, 2020; Gamella et al. 2013, 2014a, 2014b). *Gitanos*' identity often shows elements of an "oppositional identity" built in opposition or in contrast to the dominant majority culture and associated with the status of involuntary minority (Ogbu and Simons 1998). But *Gitanos* have contributed much to Spanish culture and folklore. Perhaps in no other part of Europe has such a cultural fusion occurred as in Spain, especially in Andalusia, where many of the symbols

and practices that identify the region to the world (such as flamenco singing and dancing) have a crucial *Gitano* component (Leblon 2003; Pasqualino 1998).

Almost all Spanish *Gitanos* are sedentary; they have lived in the same towns and counties for generations and often have a strong attachment to their places of birth or residence, defining themselves as Andalusians, Catalans, or even *Sevillanos* and *Granadinos*. Informed estimates of the size of the *Gitano* population put it in the range of 500,000 to 600,000, around 1.5% of the total Spanish population (FSG 2008). Although in some locations, mainly in the southern region of Andalusia where about 40% of the Spanish *Gitanos* live (even though Andalusia has less than 20% of the total Spanish population), *Gitanos* represent a particularly high fraction of the population. We conducted our study in an area of eastern Andalusia. This geographical area was chosen due to its high concentration of *Gitanos*, thus allowing the recruitment of a sufficient number of members of this ethnicity for our study. In the five towns hosting the experiments, *Gitanos* account for about 25.6% of the population on average (range: 20.0%–41.4%), that is, about 3,970 over a total of 15,490 inhabitants according to our estimates for 2007.

Some *Gitano* cultural traits are essential for understanding their social behaviour and peer punishment in particular. Such traits are mainly associated with social organization and gender roles. We summarise their differential characteristics in the following lines and develop hypotheses about how some of these cultural traits might translate, as proximate-level explanations, into observed behaviour in the experiment.

Social organization and "the family"

Even considering the growing heterogeneity of *Gitanos*, their social universe is largely based on kinship and marriage relations. Their main social networks are family networks, which tend

to be larger, denser, and more complex and multifunctional than those of their non-*Gitano* neighbors (or *Payos*, as *Gitanos* often refer to them).

For *Gitanos* today, their most important institution is “the family.” The particular notion of family among the *Gitano* population encompasses many different meanings, which can be summarised across two levels. First, compared to non-*Gitanos*, *Gitanos* display relatively smaller stress in the household or co-resident domestic unit and a more general understanding of the “closest family” as including a network of households formed by close kinship links. Considering the different moments in the developmental cycle of domestic units, it is possible to find, for instance, that a specific couple and their children gravitate heavily and almost daily towards the husband’s parents. Thus, a patri-virilocal bias strengthens the patrilineal ideology sustained primarily by males (Gay Blasco 1999; Martín and Gamella 2005; Gamella and Martín 2007; Gamella 2011). Second, kin networks include a larger number of people due to several processes that differ from the majority at large: in particular, (i) higher fertility leading to a larger number of siblings and, in turn, aunts-uncles, cousins, second cousins, etc.; and (ii) higher consanguinity in marriage that generates a multiplicity of links between members of any network, as well as higher network homogeneity, although in the last decades the heterogeneity of *Gitano* families may be increasing (Gamella and Álvarez-Roldán 2021).

Consanguinity in marriage has indeed been strikingly common among *Gitanos*, who show a marked preference to marry “known,” compatible, and “good” people from reliable interrelated kin networks. This does not stem only from geographic isolation or inheritance rules and patrimonial strategies. Rather, it is more the result of social isolation or segregation, as well as a marked cultural preference for endogamy (Gamella 2020).

It has long been argued that in premodern or “traditional” societies kinship “provides […] an organising medium of trust relations.” As such, “kinspeople can usually be relied upon to meet

a range of obligations more or less regardless of whether they feel personally sympathetic towards the specific individuals involved", while in modern societies relationships of trust have been replaced by "friendship or sexual intimacy as a means of stabilising social ties" (Giddens 1990:101–102). The dominant idea is that modernity implies isolation from kin networks and individuals confront each other as separate entities "divorced from their kinship and family units" (Finkler et al. 2001:236). This varies across countries, however (Schulz et al. 2019). Precisely, Spain as well as other southern European countries are usually portrayed as "familial" societies, where family bonds and support are relatively prominent, and individualism is somehow limited by family obligations (Reher 1998). Therefore, the distinction between *Gitanos* and Spaniards at large in this regard might be considered as a question of degree rather than as an absolute one. But the density and intensity of kin bonds often generate a differential institutional setup and affect the interpretative lens shared by local *Gitanos*.

Consanguineous marriage is much more common among *Gitanos* than among Spaniards at large and has shown both a distinctive character and evolution. Although Spain once had some of the highest levels of consanguinity in Europe, it began to fall in the 1950s and, in following decades, the fall was so rapid that consanguineous marriages have become as rare as in other Western countries (Fuster and Colantonio 2002, 2004; Calderón et al. 2009). Within *Gitano* communities, however, they have been and remain widespread. According to recent estimates based on genealogical reconstruction for the period 1925–2006 (Gamella 2020), in 22 contiguous localities in the area where this study was conducted more than half (54.8%) of all *Gitano* marriages are among relatives, with close-kin consanguineous marriages (up to second cousins) averaging 28.7%. An estimation that can be compared to the measures reported in studies using interviews or other synchronic research methods yields average inbreeding coefficients (Wright's F) of about 11.3 ($x10^{-3}$), levels never found in Spain and much less so recently. This value is rather conservative, however, and may underestimate the actual F by

more than 30% in this population. These are among the highest rates of consanguinity found in any European population, including the most inbred of Spanish isolates (Gamella 2020). In the same area, aggregate consanguinity rates for the overall population (including *Gitanos* and non-*Gitanos*) reached a maximum of around 7.4% between 1920 and 1936, with corresponding F coefficients ranging from 2.4 to 2.7 (x10^{-3}). Since the 1960s, the rates of consanguinity have decreased rapidly (Gamella and Núñez-Negrillo 2019). Note that recent comparable estimates for small-scale societies of hunter-gatherer and horticulturalists report average F values well below 2 (x10^{-3}) and 10 (x10^{-3}), respectively (Walker 2014; Walker and Bailey 2014). Given the strong correlation between coefficients of inbreeding and mean relatedness (Hamilton's r) of groups (Walker 2014), these data demonstrate that Romani people of this area are highly genetically related on average, even compared with people from small-scale societies. Multiple consanguinity is the norm among *Gitanos*: couples are linked by several bonds and share many ancestors; a product of a pattern sustained over many generations. However, these patterns are changing and the rate of intermarriage between *Gitanos* and non-*Gitanos* is increasing, particularly in some local communities (Gamella and Álvarez-Roldán 2021).

In sum, even in a region where consanguineous marriages had been important, kin endogamy among *Gitanos* shows a particularly high intensity and permanence, as it is the product of a strong cultural preference and not only of geographical isolation and poverty. Hence, it is somehow reasonable that *Gitanos* spread that sense of kin to the whole community: "here we all are family"; "all *Gitanos* are related, they share some blood, at least a drop of blood for sure"; "distant but relatives". Neighbors, friends, and partners are often family as well.

The enforcement of norms—a norm-psychology hypothesis

Regarding norm-enforcement institutions, some Romani groups have formal conflict resolution processes and tribunals. *Gitano* people, however, use more informal systems of justice and

adjudication of rights to avoid the escalation of violence and blood feuds (San Román 1986, 2010). Respected elders, typically men (*hombres de razón* or *hombres de respeto*: "men of reason" or "men of respect"), are often asked to mediate. Affinal kin relationships may also limit the extent and seriousness of conflicts, which have been recurrent and feared. Still today a serious conflict (a death) may imply the abandonment of their residences by several hundred of the closest kin of the accused.

Notwithstanding, both male and female *Gitanos*, but in different socio-political spheres, display a comparatively strong sense of individual autonomy (Gamella 2000, 2011) which, added to the possibility of escalation of conflict between families, may restrict the role of decentralised overt sanctioning unless key norms are transgressed (Piasere 2012; Matras 2015; San Román 2010; Gay Blasco 1999; Álvarez-Roldán et al. 2018). This culture of liberty or resistance, possibly related to the avoidance of conflict between *Gitano* families, should be associated with a low willingness to punish in homogeneous groups if cultural differences are translated into game play as predicted by a norm-psychology account. An earlier study with a sample of Spanish *Gitanos* provides preliminary support for this prediction. Brañas-Garza et al. (2006) used ultimatum game experiments to examine sharing and punishment behaviour in anonymous one-shot bilateral interactions between *Gitanos* in Vallecas, Madrid. Most of them did not express any willingness to punish stingy co-ethnics (but see Espín et al. 2012, 2015 for combined evidence suggesting that the psychology underlying the rejection of low offers in the ultimatum game may differ from that underlying altruistic punishment in the PGP). Furthermore, a common rationale of *Gitanos* who were unwilling to reject unfair, even zero, offers was, "What if (s)he needs the money?". This suggests that sporadic acts of uncooperativeness carried out by *Gitanos* may not per se be considered by other *Gitanos* as deserving peer punishment; solidarity and forgiveness might be the intuitive response.

Gender roles—a norm-psychology hypothesis

In general, *Gitanos* are portrayed as a group that sustains relatively conservative or patriarchal gender relationships, where women are subordinated to fathers and brothers when they are single, and to their husbands and husband's family when married (San Román 2010; Gay Blasco 1999). Care of children, family members, and the sick are generally seen as women's primordial tasks, but in this regard there is only a degree of difference with non-*Gitanos* of this area.

However, the considerable agency developed by *Gitano* women in their daily lives, both in the domestic and public realms, is rarely considered. It is often *Gitano* women who confront authorities in administrative matters and in the defense of their rights to housing, education or public benefits. But they do that somehow as in delegation by their husbands and partners; it is part of their accepted gender roles. In confrontational encounters judged as impersonal, *Gitano* women can be very assertive and their attitudes are often seen as inadequate by majority standards, as if they were not following the same patterns of modesty and good manners of middle-class Spaniards (Gamella 2000, 2011). This supposed lack of accommodation to their subordinate status is part of the generalised anti-*Gitano* bias that reflects important majority norms; a process also found with respect to anti-Roma bias in Eastern Europe (Kende et al. 2017).

But in personal interactions, or in front of *Gitano* people, the presence of males in public encounters somehow transforms the ways most *Gitano* women will voice their concerns and pursue their interests. There exists a number of principles that *Gitano* women must typically follow in these cases: e.g., "never let him lose face in public" or "never contradict him or the elders publicly". If women decide or influence family decisions, as they often do, their role has to be more private than public, more by applying reason than violence (Gamella 2000; Gamella and Martín 2007). In this sense, while gossiping is a fundamental weapon in the hands of women, violence is seen as the prerogative of males in extreme circumstances (Gay Blasco

1999; San Román 2010). There is obviously much variation among individuals and couples in these gender arrangements and age may also play an important moderating role, but this norm clearly differs with respect to the majority population. Following the norm-psychology account, this cultural difference is hypothesised to be reflected in game behaviour in that *Gitano* females should be more reluctant (than non-*Gitano* ones and males in general) to punish others in either condition of the experiment given that *Gitano* males are always present.

References

Álvarez-Roldán, A., Parra, I., & Gamella, J. F. (2018). Reasons for the underachievement and school drop out of Spanish Romani adolescents. A mixed methods participatory study. *International Journal of Intercultural Relations*, *63*, 113-127.

Brañas-Garza, P., Cobo-Reyes, R., & Domínguez, A. (2006). “Si él lo necesita”: Gypsy fairness in Vallecas. *Experimental Economics*, *9*(3), 253-264.

Calderón, R., Aresti, U., Ambrosio, B., & González-Martín, A. (2009). Inbreeding Coefficients for X-linked and Autosomal Genes in Consanguineous Marriages in Spanish Populations: The Case of Guipúzcoa (Basque Country). *Annals of Human Genetics, 73*(2), 184-195.

Cantón, M. (2010) “Gypsy Pentecostalism, ethnopolitical uses and construction of belonging in the south of Spain”. *Social Compass (International Review of Sociology of Religion)*, Vol. 57, nº 2, pp. 253-267.

Cantón, M. (2020). *Evangelical Gypsies in Spain:" The Bible is our Promised Land"*. Lexington: Lexington Books.

Espín, A. M., Brañas-Garza, P., Herrmann, B., & Gamella, J. F. (2012). Patient and impatient punishers of free-riders. *Proceedings of the Royal Society of London B: Biological Sciences*, rspb20122043.

Espín, A. M., Exadaktylos, F., Herrmann, B., & Brañas-Garza, P. (2015). Short-and long-run goals in ultimatum bargaining: impatience predicts spite-based behavior. *Frontiers in Behavioral Neuroscience*, *9*, 214.

Finkler, K., Dolgin, J., Franklin, S., Gusterson, H., Hadler, N. M., Evans, J. P., ... & Finkler, K. (2001). The kin in the gene: the medicalization of family and kinship in American society. *Current Anthropology*, *42*(2), 235-263.

Fraser, A. (1992). *The Gypsies: The people of Europe*. Oxford: Blackwell Publishers.

FSG (2008) *Mapa sobre vivienda y comunidad gitana en España, 2007*. Madrid: Fundación Secretariado Gitano.

Fuster, V., & Colantonio, S. (2004). Socioeconomic, demographic, and geographic variables affecting the diverse degrees of consanguineous marriages in Spain. *Human Biology*, *76*(1), 1-14.

Fuster, V., & Colantonio, S. E. (2002). Consanguinity in Spain: socioeconomic, demographic, and geographic influences. *Human Biology*, 301-315.

Gamella, J. F. (2000). *Mujeres gitanas: matrimonio y género en la cultura gitana de Andalucía*. Junta de Andalucía.

Gamella, J. F. (2011). *Historias de éxito. Modelos para reducir el abandono escolar de la adolescencia gitana*. Madrid: IFIIE, Ministerio de Educación.

Gamella, J. F., & Álvarez-Roldán, A. (2021). Breaking secular endogamy. A case study of the growth of intermarriage among the Gitanos/Calé of Spain. *Mimeo*.

Gamella, J. F., Carrasco-Muñoz, E. M., & Núñez Negrillo, A. M. (2013). Oculocutaneous albinism and consanguineous marriage among Spanish *Gitanos* or Calé–A study of 83 cases. *Collegium Antropologicum*, *37*(3), 723-734.

Gamella, J. F., Carrasco-Muñoz, E. M., & Quesada Garrido, A. (2014a). New methodological approaches in the anthropological demography of Romani groups. An example from the study of the evolution of the infant and child mortality of the *Gitanos* or Calé of Spain (1871-2007). *Sociologia: Revista da Faculdade de Letras da Universidade Do Porto*, *4*, 175-204.

Gamella, J. F., Gómez Alfaro, A., & Pérez Pérez, J. (2014b). Nominal Assimilation: The Ethnic and National Identities of the *Gitanos* or Calé of Spain as Shown by their Surnames in the 1783–1785 Census. *Names*, *62*(3), 147-164.

Gamella, J. F., Martín, E. (2007). Vien avec moi cousine. Le mariage entre cousins germains chez le Gitans Andalous. *Etudes Tsiganes*, 30, 110-151.

Gamella, J.F. & Núñez-Negrillo, A.M. (2019). The Evolution of Consanguineous Marriages in the Archdiocese of Granada, Spain (1900-1979). *Human Biology*, *90*(2): 97-114.

Gamella, J. F. (2020). Consanguineous marriages among Andalusian Gitanos/Calé: a genealogical analysis (1925–2006). *Journal of Biosocial Science*, 52(6): 809-831.

Gay Blasco, P. (1999). *Gypsies in Madrid: Sex, gender and the performance of identity*. Bloomsbury Academic.

Giddens, A. (1990). *The consequences of modernity*. Cambridge: Polity.

Gómez Alfaro A. (1999). Tipologías. Matrimonios mixtos y mestizajes gitanos en los censos históricos andaluces. *Demófilo, 30,* 30–52.

Gómez Alfaro, A. (1998). Gitanos de Priego de Córdoba: entre el acoso y la supervivencia. *Legajos. Cuadernos de Investigación Histórica del Sur de Córdoba, 1,* 61-72.

Kende, A., Hadarics, M., & Lášticová, B. (2017) Anti-Roma attitudes as expressions of dominant social norms in Eastern Europe. *International Journal of Intercultural Relations,* 60: 12-27. DOI: doi.org/10.1016/j.ijintrel.2017.06.002

Leblon, B. (1985). *Les Gitans d'Espagne*. Paris: Presses Universitaires de France.

Leblon, B. (2003). *Gypsies and flamenco: the emergence of the art of flamenco in Andalusia* (Vol. 6). Univ of Hertfordshire Press.

Martin, E., & Gamella, J. F. (2005). Marriage practices and ethnic differentiation: The case of Spanish Gypsies (1870–2000). *The History of the Family*, *10*(1), 45-63.

Matras, Y. (2002). *Romani: A linguistic introduction*. Cambridge University Press.

Matras, Y. (2015). *The Romani Gypsies*. Harvard University Press.

Mendizabal, I., Lao, O., Marigorta, U. M., Wollstein, A., Gusmão, L., Ferak, V., ... & Kučinskas, V. (2012). Reconstructing the population history of European Romani from genome-wide data. *Current Biology, 22*(24), 2342-2349.

Ogbu, J. U., & Simons, H. D. (1998). Voluntary and involuntary minorities: a cultural-ecological theory of school performance with some implications for education. *Anthropology & Education Quarterly*, *29*(2), 155-188.

Pasqualino, C. (1998). *Dire le chant. Les Gitanes flamencos d'Andalousie*. Paris: CNRS Editions.

Peiró, E. W. (2012). *White Gypsies: Race and stardom in Spanish musical films*. University of Minnesota Press.

Piasere, L. (2004). *I rom d'Europa: una storia moderna*. GLF editori Laterza.

Piasere, L. (2012). *Scenari dell'antiziganismo.* Firenze: SEID Editori.

Pym, R. (2007). *The Gypsies of early modern Spain, 1425-1783*. London: Palgrave Macmillan.

Reher, D. S. (1998). Family ties in Western Europe: persistent contrasts. *Population and Development Review, 24*(2), 203-234.

San Román, T. (1986). *Entre la marginación y el racismo. Reflexiones sobre la vida de los gitanos*. Madrid: Alianza Universidad.

San Román, T. (2010). *La diferencia inquietante: viejas y nuevas estrategias culturales de los gitanos*. Siglo XXI de España Editores.

Schulz, J. F., Bahrami-Rad, D., Beauchamp, J. P., & Henrich, J. (2019). The Church, intensive kinship, and global psychological variation. *Science, 366*(6466), eaau5141.

Walker, R. S. (2014). Amazonian horticulturalists live in larger, more related groups than hunter–gatherers. *Evolution and Human Behavior, 35*(5), 384-388.

Walker, R. S., & Bailey, D. H. (2014). Marrying kin in small-scale societies. *American Journal of Human Biology, 26*(3), 384-388.

Supplementary Figures and Tables

Figure S1*. Contribution decision card (Yellow #1 participant example; translated from Spanish)*

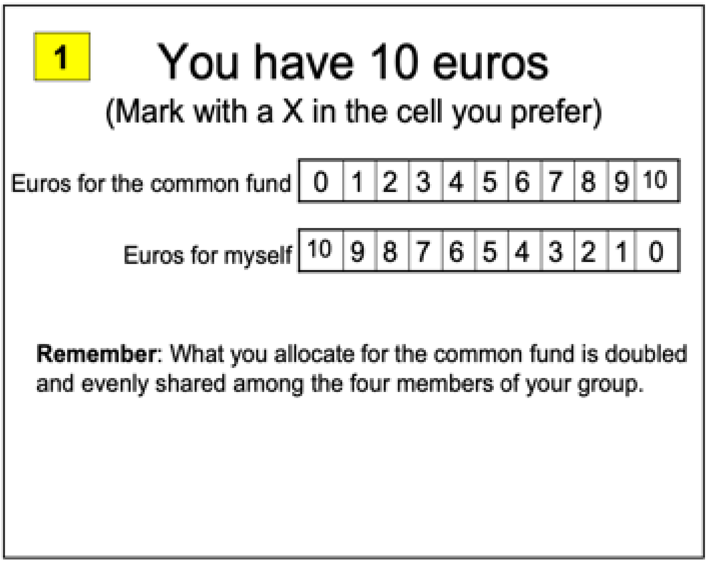

Figure S2*. Punishment decision card (Yellow #1 participant example; translated from Spanish)*

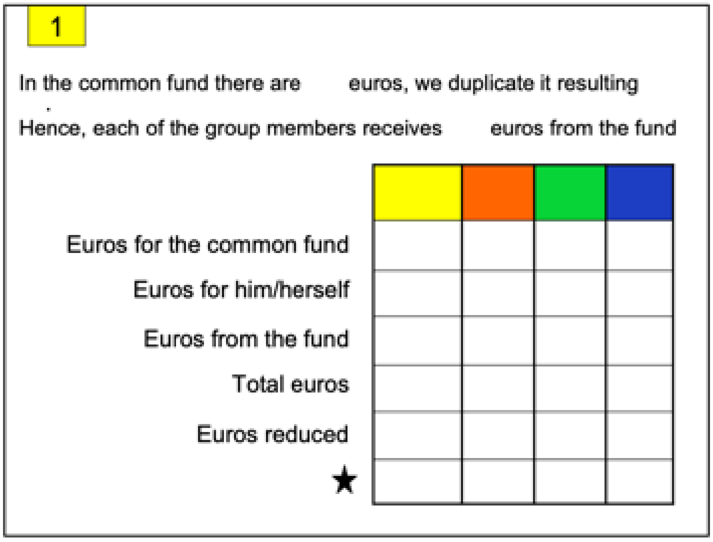

Table S1. *The determinants of contributions to the public good*

depvar: *contribution*	(1a)	(2a)	(3a)	(4a)	(5a)	(1b)	(2b)	(3b)	(4b)	(5b)
gitano	-0.361	0.003	-0.145	-0.313	0.116	-0.270	0.065	0.011	-0.248	0.217
	(0.321)	(0.456)	(0.399)	(0.319)	(0.549)	(0.417)	(0.524)	(0.488)	(0.414)	(0.601)
mixed	0.306	0.639	0.321	0.921**	1.134**	0.388	0.694	0.417	0.971**	1.174**
	(0.312)	(0.423)	(0.313)	(0.384)	(0.483)	(0.323)	(0.428)	(0.324)	(0.387)	(0.482)
male	-0.181	-0.156	0.083	0.605	0.867	-0.131	-0.110	0.225	0.626	0.976
	(0.333)	(0.336)	(0.490)	(0.486)	(0.686)	(0.342)	(0.344)	(0.500)	(0.495)	(0.709)
gitano X mixed		-0.733			-0.611		-0.682			-0.574
		(0.620)			(0.799)		(0.622)			(0.799)
gitano X male			-0.526		-0.648			-0.689		-0.819
			(0.663)		(0.983)			(0.663)		(0.996)
mixed X male				-1.543**	-1.725*				-1.491**	-1.685*
				(0.641)	(0.971)				(0.646)	(0.987)
gitano X mixed X male					0.613					0.650
					(1.336)					(1.353)
age						0.011	0.010	0.012	0.009	0.010
						(0.010)	(0.010)	(0.010)	(0.010)	(0.010)
hincome						-0.045	-0.042	-0.056	-0.045	-0.056
						(0.135)	(0.135)	(0.133)	(0.129)	(0.130)
Constant	6.380***	6.224***	6.297***	6.087***	5.941***	5.928***	5.801***	5.793***	5.732***	5.575***
	(0.262)	(0.296)	(0.278)	(0.282)	(0.322)	(0.836)	(0.840)	(0.846)	(0.825)	(0.839)
F	0.870	1.042	0.780	2.296*	1.420	0.927	1.000	0.969	1.816*	1.347
Log-likelihood	-754.844	-754.136	-754.510	-751.791	-751.350	-753.927	-753.314	-753.368	-751.077	-750.513
R^2	0.009	0.013	0.011	0.028	0.030	0.014	0.018	0.018	0.032	0.036
Obs.	313	313	313	313	313	313	313	313	313	313

Notes: OLS estimates. Robust standard errors are shown in parentheses. Dependent variable: contribution (euros; range 0–10). Main explanatory variables: *gitano*, *mixed*, *male* and their interactions are binary variables (0/1). Columns 1b–5b repeat the regressions adding control variables: *age* (range 16–-82), *hincome* (household income: range 0–9, from “0 euros/month” to “more than 5,000 euros/month”; 12 missing values were imputed using OLS regression with *gitano*, *age*, and *male* as explanatory variables). * $P < .10$, ** $P < .05$, *** $P < .01$.

Table S2. *The determinants of punishment (aggregate)*

depvar: *punishment*	(1a)	(2a)	(3a)	(4a)	(5a)	(1b)	(2b)	(3b)	(4b)	(5b)
gitano	-0.362***	-0.870***	-0.616***	-0.459***	-1.050***	-0.304**	-0.813***	-0.559***	-0.382**	-0.962***
	(0.116)	(0.156)	(0.122)	(0.114)	(0.173)	(0.153)	(0.162)	(0.155)	(0.149)	(0.184)
mixed	-0.065	-0.418**	-0.080	-0.492***	-0.748***	-0.052	-0.403**	-0.075	-0.477***	-0.738***
	(0.148)	(0.193)	(0.145)	(0.158)	(0.221)	(0.152)	(0.193)	(0.150)	(0.159)	(0.223)
male	0.134	0.120	-0.147	-0.373***	-0.656***	0.126	0.119	-0.166	-0.386***	-0.668***
	(0.118)	(0.124)	(0.189)	(0.136)	(0.186)	(0.123)	(0.130)	(0.198)	(0.142)	(0.193)
differ	0.074***	0.076***	0.073***	0.078***	0.078***	0.074***	0.076***	0.073***	0.077***	0.078***
	(0.017)	(0.017)	(0.017)	(0.016)	(0.017)	(0.017)	(0.017)	(0.017)	(0.017)	(0.017)
meancont2others	0.077**	0.070**	0.080***	0.073**	0.073**	0.077***	0.070**	0.080***	0.074***	0.073***
	(0.030)	(0.030)	(0.030)	(0.029)	(0.028)	(0.030)	(0.029)	(0.029)	(0.028)	(0.028)
gitano X mixed		0.807***			0.730***		0.814***			0.738***
		(0.228)			(0.242)		(0.230)			(0.244)
gitano X male			0.574**		0.700***			0.579**		0.690***
			(0.231)		(0.241)			(0.233)		(0.249)
mixed X male				1.085***	1.165***				1.113***	1.192***
				(0.223)	(0.358)				(0.224)	(0.355)
gitano X mixed X male					-0.476					-0.488
					(0.434)					(0.438)
age						0.002	0.003	0.001	0.004	0.004
						(0.005)	(0.005)	(0.005)	(0.005)	(0.005)
hincome						0.019	0.014	0.027	0.016	0.024
						(0.043)	(0.044)	(0.044)	(0.044)	(0.045)
Constant	0.396*	0.652***	0.474**	0.636***	0.848***	0.224	0.458	0.326	0.366	0.585*
	(0.234)	(0.246)	(0.230)	(0.224)	(0.239)	(0.325)	(0.318)	(0.320)	(0.319)	(0.320)
Chi^2	38.045***	52.992***	53.771***	58.644***	78.609***	40.733***	55.917***	54.738***	64.577***	80.230***
Log-likelihood	-1712.275	-1707.827	-1709.767	-1703.265	-1698.768	-1712.103	-1707.575	-1709.597	-1702.715	-1698.316
R^2	0.048	0.066	0.059	0.082	0.095	0.049	0.067	0.059	0.084	0.097
Obs.	939	939	939	939	939	939	939	939	939	939

Notes: GLMM random effects estimates. Dependent variable: punishment (euros reduced per target; range 0–9). Main explanatory variables: same as in Table S1 + *differ* (punisher's contribution – target's contribution, from -10 to 10) + *meancont2others* (mean contribution of other 2 group members, range 0–10). See notes in Table S1.

Table S3. *The determinants of punishment (mixed groups)*

depvar: *punishment*	(1a)	(2a)	(3a)	(4a)	(5a)	(1b)	(2b)	(3b)	(4b)	(5b)
gitano	-0.140	-0.312	-0.141	-0.139	-0.356*	-0.103	-0.275	-0.101	-0.104	-0.317
	(0.173)	(0.219)	(0.173)	(0.174)	(0.216)	(0.240)	(0.270)	(0.240)	(0.243)	(0.271)
male	0.579***	0.579***	0.572***	0.583***	0.572***	0.656***	0.656***	0.648***	0.664***	0.651***
	(0.179)	(0.179)	(0.177)	(0.178)	(0.175)	(0.181)	(0.181)	(0.179)	(0.180)	(0.178)
targetgit	0.103	-0.072	0.102	0.098	-0.103	0.104	-0.071	0.103	0.099	-0.103
	(0.151)	(0.258)	(0.151)	(0.150)	(0.256)	(0.151)	(0.258)	(0.151)	(0.150)	(0.255)
differ	0.096***	0.096***	0.084**	0.044*	0.030	0.094***	0.094***	0.081**	0.041*	0.024
	(0.022)	(0.022)	(0.034)	(0.023)	(0.046)	(0.022)	(0.022)	(0.034)	(0.022)	(0.044)
meancont2others	0.051	0.052	0.054	0.048	0.052	0.054	0.054	0.056	0.050	0.054
	(0.042)	(0.042)	(0.044)	(0.038)	(0.040)	(0.040)	(0.040)	(0.042)	(0.037)	(0.038)
gitano X targetgit		0.342			0.411		0.342			0.411
		(0.321)			(0.322)		(0.321)			(0.322)
gitano X differ			0.024		0.018			0.024		0.024
			(0.045)		(0.052)			(0.043)		(0.052)
targetgit X differ				0.105***	0.091**				0.106***	0.096**
				(0.037)	(0.046)				(0.036)	(0.046)
gitano X targetgit X differ					0.057					0.049
					(0.074)					(0.078)
age						0.006	0.006	0.006	0.006	0.006
						(0.007)	(0.007)	(0.007)	(0.007)	(0.008)
hincome						-0.029	-0.029	-0.028	-0.031	-0.030
						(0.055)	(0.055)	(0.055)	(0.055)	(0.054)
Constant	0.131	0.246	0.127	0.127	0.257	-0.087	0.028	-0.098	-0.091	0.037
	(0.305)	(0.330)	(0.310)	(0.290)	(0.319)	(0.494)	(0.509)	(0.497)	(0.508)	(0.522)
Chi^2	29.914***	34.469***	33.038***	39.045***	46.292***	31.538***	37.257***	34.802***	43.635***	53.476***
Log-likelihood	-857.328	-856.552	-857.163	-853.233	-850.976	-856.623	-855.847	-856.455	-852.468	-850.243
R^2	0.081	0.083	0.080	0.095	0.102	0.091	0.093	0.091	0.106	0.113
Obs.	465	465	465	465	465	465	465	465	465	465

Notes: GLMM random effects estimates. Dependent variable: punishment (euros reduced per target; range 0–9). Main explanatory variables: same as in Table S2 + *targetgit* (binary variable: whether the target is gitano (0/1)). See notes in Table S1.
